# Post‐Seismic Deformation Related to the 2016 Central Italy Seismic Sequence From GPS Displacement Time‐Series

**DOI:** 10.1029/2021JB022200

**Published:** 2021-08-29

**Authors:** E. Mandler, F. Pintori, A. Gualandi, L. Anderlini, E. Serpelloni, M. E. Belardinelli

**Affiliations:** ^1^ Dipartimento di Fisica e Astronomia “Augusto Righi” Alma Mater Studiorum Università di Bologna Bologna Italy; ^2^ Istituto Nazionaledi Geofisica e Vulcanologia (INGV) Osservatorio Nazionale Terremoti Roma Italy; ^3^ Istituto Nazionale di Geofisica e Vulcanologia (INGV) Sezione di Bologna Bologna Italy

## Abstract

The 2016–2017 Central Italy earthquake sequence struck the central Apennines between August 2016 and October 2016 with Mw ∈ [5.9; 6.5], plus four earthquakes occurring in January 2017 with Mw ∈ [5.0; 5.5]. We study Global Positioning System time series including near‐ and far‐field domains. We use a variational Bayesian independent component analysis technique to separate the post‐seismic deformation from signals caused by variation of the water content in aquifers at hundreds of meters of depth and of the soil moisture. For each independent component, realistic uncertainties and a plausible physical explanation are provided. We focus on the study of afterslip on the main structures surrounding the mainshock, highlighting the role played by faults that were not activated during the co‐seismic phase in accommodating the post‐seismic deformation. We report aseismic deformation occurring on the Paganica fault, which hosted the Mw 6.1 2009 L'Aquila earthquake, suggesting that static stress transfer and aseismic slip influence the recurrence time of nearby (∼50 km further south of the mainshocks) segments. A ∼2–3 km thick subhorizontal shear‐zone, clearly illuminated by seismicity, which bounds at depth the west‐dipping normal faults where the mainshocks nucleated, also shows aseismic slip. Since afterslip alone underestimates the displacement in the far‐field domain, we consider the possibility that the shear zone marks the brittle‐ductile transition, assuming the viscoelastic relaxation of the lower crust as a mechanism contributing to the post‐seismic displacement. Our results suggest that multiple deformation processes are active in the first 2 years after the mainshocks.

## Key Points

- We study the post‐seismic deformation following the 2016–2017 Central Italy seismic sequence through Global Positioning System displacement time series
- We map the afterslip distribution on faults co‐seismically activated and we investigate interaction among faults
- We infer the occurrence of multiple post‐seismic deformation mechanisms including afterslip and viscoelastic relaxation

## 1 Introduction

**Figure 1:**
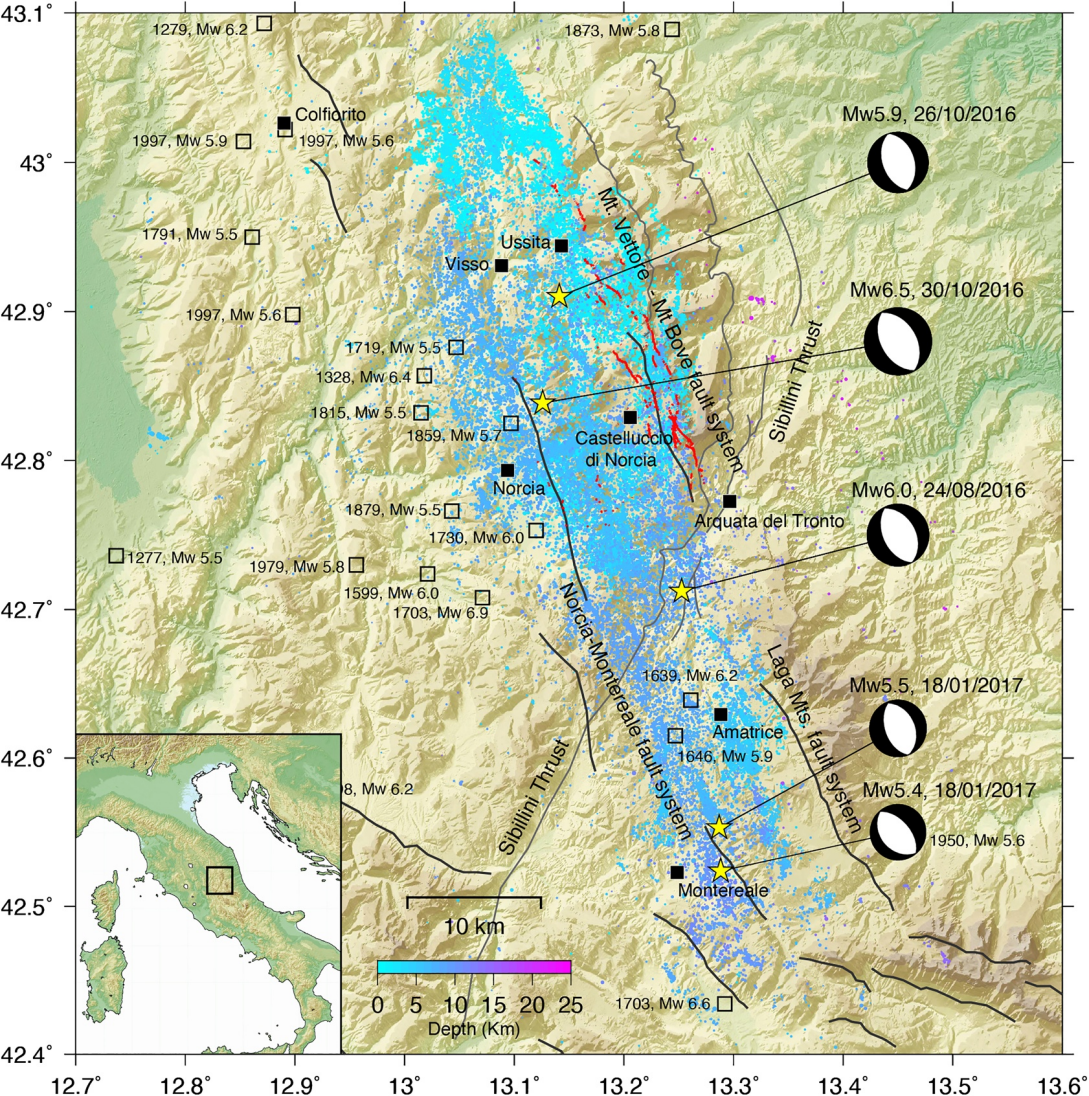
Map showing the major events of the 2016–2017 Central Italy sequence (yellow stars), the focal mechanism (from Michele et al., 2020) and the historical seismicity since year 1005 (squares), for earthquakes with equivalent 5.4 ≤ Mw (from CPTI15, V.2.0, https://emidius.mi.ingv.it/CPTI15-DBMI15). Colored dots represent the seismicity recorded after August 24 (from Michele et al., 2020), plotted as a function of depth. The red lines represent ground ruptures associated with the Amatrice and Norcia mainshocks (from Civico et al., 2018). The black and gray lines show traces of major normal faults and of the Sibillini thrust, respectively.

The 2016 Amatrice‐Visso‐Norcia earthquake sequence started on August, 24 when a Mw 6.0 event struck a sector of the Central Apennines (Figure 1) that is characterized by a narrow band of measurable geodetic and seismic deformation rates (Barani et al., 2017; D'Agostino, 2014; Sani et al., 2016). It caused hundreds of deaths and considerable damage to the town of Amatrice and its surroundings (Pucci et al., 2017; Figure 1). Seismicity followed the mainshock both northwest and southeast of the epicenter (Chiaraluce, Di Stefano, et al., 2017) with decreasing magnitude and frequency, until when a Mw 5.9 event occurred on October 26, about 25 km to the NW of Amatrice's earthquake epicenter, near the village of Visso (Figure 1). On October 30, the largest event of the seismic sequence (Mw 6.5) occurred near the town of Norcia, involving a portion of the fault system between the two preceding events which had previously been left unruptured (Cheloni et al., 2017). The seismic sequence continued on January 18, 2017 with four 5 ≤ Mw ≤ 5.5 earthquakes that ruptured the Campotosto fault, southeast of the Amatrice event (Cheloni et al., 2017; Xu et al., 2017).

The main events show normal faulting mechanisms (http://cnt.rm.ingv.tdmt; Figure 1) in agreement with the SW‐NE extension of ∼3–4 mm/year that characterizes this area (Barani et al., 2017; D'Agostino, 2014; Devoti et al., 2017), and with the presence of several NW‐SE trending active normal faults aligned along the Apennines chain (Boncio et al., 2004; Galli et al., 2008; Pizzi & Galadini, 2009). The bulk of geological (e.g., Civico et al., 2018; EMERGEO Working Group, 2016; Falcucci et al., 2016; Galadini et al., 2018; Pizzi et al., 2017; Villani et al., 2018), seismological (Chiaraluce, Di Stefano, et al., 2017; Papadopoulos et al., 2017; Pizzi et al., 2017; Scognamiglio et al., 2018; Tinti et al., 2016), and geodetic (Cheloni et al., 2017; Huang et al., 2017; Lavecchia et al., 2016; Walters et al., 2018; Wang et al., 2018; Xu et al., 2017) observations, collected soon after the Amatrice mainshock, agree in showing that each mainshock broke different, slightly off‐axis, segments of a SW dipping normal fault system. Moving from north to south two main normal fault segments can be detected, respectively the Mt. Vettore‐Mt. Bove and Mt. della Laga fault (also known as the Gorzano fault), separated by the Pliocene Sibillini thrust (Figure 1).

The area has been repeatedly struck by 5.2 < Mw < 6.2 earthquakes in the last 400 years (Rovida et al., 2019; Figure 1). Fault segments responsible for the mainshocks of this seismic sequence involved a portion of a fault system as long as 80 km along strike (Figure 1; Michele et al., 2020). Importantly, the 2016 Central Italy sequence is bounded to the north by the fault system responsible for the Colfiorito 1997 seismic sequence (Amato et al., 1998; Boncio & Lavecchia, 2000; Chiaraluce et al., 2003; Ferrarini et al., 2015), and to the south by the one responsible for the 2009 L'Aquila earthquake (Chiaraluce, 2012; Lavecchia et al., 2012; Valoroso et al., 2013). Therefore these recent earthquake sequences can be interpreted in the light of a 150 km long normal fault system, made up of 10–30 km long segments, separated by crosscutting compressional structures inherited from the pre‐Quaternary compressional tectonics (Pizzi & Galadini, 2009).

The comparison between the subsurface geology and seismological data (e.g., Porreca et al., 2018) shows that most of the instrumental background seismicity recorded after the 1997, the 2009, and the 2016 seismic sequences is confined within the sedimentary succession that overlays the Dolostones, which is the characteristic lithology of this area (Chiaraluce, Barchi, et al., 2017; Chiaraluce, Di Stefano, et al., 2017). Importantly, the downdip extension of the normal fault system is limited within the first 8–10 km of the upper crust, being bounded at depth by a gently east‐dipping, ∼2–3 km thick, layer of seismicity that likely decouples the upper and lower crust (Chiaraluce, Di Stefano, et al., 2017). This hosted a series of small to moderate aftershocks (≈Mw 4) being possibly involved in the loading of the higher angle faults above (Vuan et al., 2017). Following previous authors (e.g., Chiaraluce, Di Stefano, et al., 2017; Michele et al., 2020; Vuan et al., 2017), we refer to such a layer of seismicity as the “shear zone.”

It is well known that after moderate size earthquakes, like the ones here studied, post‐seismic displacements are recorded at the surface. There are typically multiple possible candidate mechanisms to explain the post‐seismic geodetic observations: afterslip on the surrounding of the mainshock (e.g., Perfettini & Avouac, 2007), viscoelastic relaxation of the lower crust/upper mantle (e.g., Pollitz et al., 2000), and poroelastic flow due to fluid redistribution after the stress variations imparted by the mainshock in the crust (e.g., Fialko, 2004). To the best of our knowledge, the only published study concerning the post‐seismic deformation following the 2016 Central Italy sequence is the one of Pousse‐Beltran et al. (2020). In that study, the focus was mainly on the early (i.e., first 6 months) and near field (<50 km) response, and for this reason, they invoked only afterslip to model the observations. In this work, we use ground displacement time series obtained from the analysis of GPS stations, and since earthquakes of magnitude *M* > ∼6 may generate viscoelastic relaxation (e.g., Bruhat et al., 2011), here we consider a wider region (up to 100 km) and a longer time span (up to 2.3 years) in order to investigate, beside afterslip, the potential role of this further deformation mechanism. Recognizing such a process in the data allows us to constrain the rheological properties of the crust and mantle. Given the spatial extent of our data distribution, we test the hypothesis that afterslip occurred not only on the faults involved during the coseismic stage, but also on further structures like the one where the 2009 L'Aquila earthquake nucleated (i.e., the Paganica fault) and the sub‐horizontal shear zone. A possible role of this latter structure, together with a viscoelastic relaxation of the lower crust, were only hinted by Pousse‐Beltran et al. (2020) to improve the fit to the available post‐seismic deformation measurements. In doing this, we follow the principle of seeking the simplest solution capable of describing the observed post‐seismic displacement field. Moreover we apply a blind‐source‐separation algorithm to characterize the temporal evolution and spatial features of the post‐seismic deformation signal across the 2016–2017 epicentral area, separating tectonic sources from non tectonic ones. In fact, geodetic data are also affected by non‐tectonic deformation sources, and to properly interpret the observations, it is crucial to disentangle the various contributions.

The remaining of the paper is organized as follows: in Section 2, we describe the GPS data set used and the results of the independent component analysis (ICA) applied to the displacement time‐series; in Section 3, we interpret the non tectonic signals as due to hydrological sources detected by the ICA; in Section 4, we analyze the post‐seismic deformation, modeling it by inverting the GPS time series for afterslip and discussing a possible viscoelastic contribution to the measured geodetic displacements; in Section 5, we discuss the findings of this study and in Section 6, conclusions are drawn.

## 2 GPS Data and Time‐Series Analysis

### 2.1 Data

**Figure 2:**
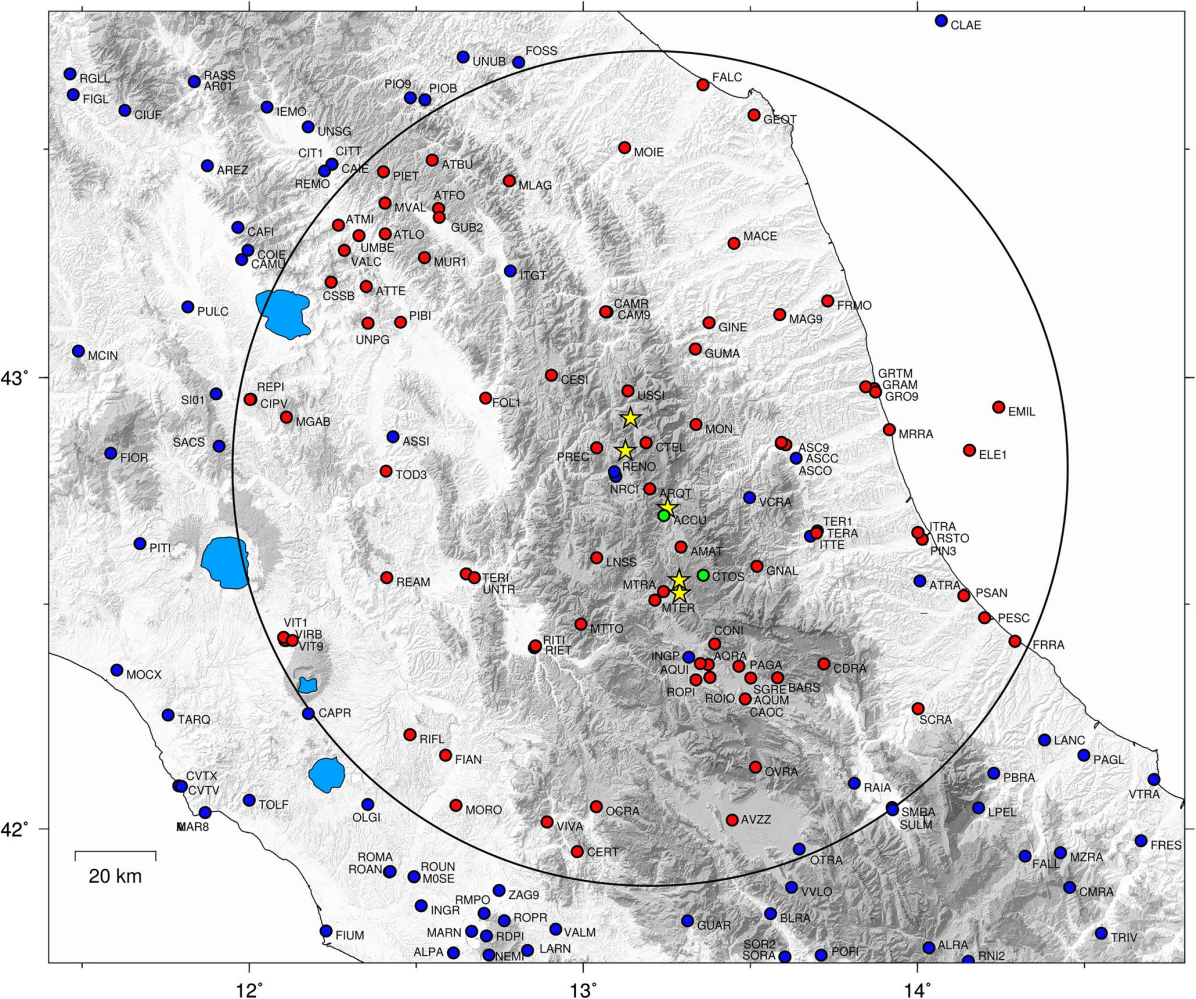
Colored circles show the Global Positioning System (GPS) stations considered. The blue circles show the positions of the continuous GPS stations present in this area and for which we analyze the raw data as described in Section 2.1. The red circles show the position of the continuous GPS stations used in the blind source separation analysis with the variational Bayesian ICA (vbICA) method (Section 2.2), namely stations within a radius of 100 km from the epicentral area having almost continuous observations after the Amatrice earthquake. The green circles show the position of the two non‐permanent GPS stations, belonging to the CaGeoNet network, included also in the vbICA. The yellow stars show the epicenters of the mainshocks of the 2016–2017 seismic sequence, as in Figure 1.

We use in total 85 GPS stations in this work (Figure 2) with almost continuous data in the time‐interval 2012–2019 integrated by a few campaign‐mode stations, belonging to the CaGeoNet network (Galvani et al., 2013), that have been occupied almost continuously after the Amatrice mainshock (see Cheloni et al., 2016 for details). The data set also includes few stations in the Adriatic on‐shore and off‐shore (Palano et al., 2020), managed by the private company ENI S.p.A. (https://www.eni.com), and new continuous stations installed as emergency response soon after the Amatrice and Norcia mainshocks.

The position time‐series have been obtained following the procedure described in Serpelloni et al. (2006, 2013, 2018), consisting of: raw phase data reduction; combination of loosely constrained network solutions and definition of the reference frame; time‐series analysis, including velocity estimates, spatial filtering of common mode errors and co‐seismic and instrumental offsets removal. The details of the processing and post‐processing procedures are described in the supporting information (Section S1.1). The time‐series used in this work are part of a continental‐scale geodetic solution including >3,500 continuous GPS stations and the spatial filtering has been applied at a continental‐scale, following Serpelloni et al. (2013, 2018), excluding all GPS stations affected by earthquakes, thus preventing the removal of the localized geophysical signals recorded by the GPS stations in the study area.

Hence, our observations consist of surface displacements that are the result of multiple processes active at the same time at different spatial and temporal scales. The proper modeling of these various contributions is an active research domain of solid Earth sciences. In practice, we aim at isolating the deformation signals relative to the various mechanisms contributing to the observations. In this sense, we are facing a so‐called blind source separation problem, as we will detail in Section 2.2. In Section 2.3, we will present the results of such analysis, and we also try to detect possible pre‐seismic signals acting prior to the Amatrice earthquake.

### 2.2 Blind Source Separation

Given a certain number *M* of displacement time series relative to sensors distributed at different locations in space, we can organize the data into a spatio‐temporal matrix *X*. In particular, we organize it in such a way that each row is a different time series, and each column represents the record at a given time or epoch. The data at our disposal has a daily sampling in time and spans the time range 2012–2019. The total number of stations used in the analysis is 85. Since we have three‐dimensional records (east, north, and vertical), the matrix *X* has size *M* × *T* = 255 × 2525. A well‐established approach to deal with the separation of the sources contributing to the observed deformation consists in the application of multivariate statistical techniques that attempts to maximize the independence of the sources generating the observations. The techniques fall under the umbrella of the ICA and typically consist in a linear decomposition of the data into a mixing matrix *A* (*M* × *L*) and a source matrix *S* (*L* × *T*), with the *L* sources (components) being as much independent as possible. We can cast the problem in the following terms:

(1) X=AS+N

where *N* is noise (typically Gaussian). Given the data *X* the goal is to find the linear mix of independent sources that generated the observations. We underline here that the equality holds if we use a number of components *L* sufficient to span the whole original space where the data *X* lives. A truncation is typically performed, that is, *L* < min{*M*,*T*}, and the right hand side is a low‐rank approximation of the left hand side (e.g., Kositsky & Avouac, 2010). Moreover, we are performing a linear decomposition of the spatio‐temporal matrix, that is, the spatial and temporal information can be splitted and encoded in the matrices *A* and *S*, respectively. Each row of *S* contains a different source. The ICA attempts to find these sources imposing them to be as statistically independent as possible while still fitting the data. Given the way we have built *X*, we are performing the decomposition in so‐called T‐mode, that is, we attempt to find sources that are independent in the time domain. We underline here that one of the strengths of these techniques consists in the fact that they are data driven and do not impose any specific prescribed functional form to the underlying sources that we want to investigate. In fact, ICA techniques belong to the so‐called unsupervised learning approaches to pattern recognition.

Unfortunately, the independence condition is not straightforward to impose and approximations are introduced in order to generate a suitable cost function to minimize or maximize. To achieve this, several approaches have been proposed and here we use a variational Bayesian ICA (vbICA). It has been shown that this method is superior to other widely used ICA techniques because it offers more flexibility in the description of multimodal sources and it allows us to take into account missing data (e.g., Chan et al., 2003; Roberts & Choudrey, 2003). We use the version adapted to the study of geodetic time series by Gualandi, Avouac, et al. (2016) and Gualandi, Serpelloni, and Belardinelli (2016). We adopt a notation similar to that used in Gualandi, Avouac, et al. (2016) and Gualandi, Serpelloni, and Belardinelli (2016), where each IC is characterized by a specific spatial distribution (*U*) and a temporal evolution (*V*). A weight coefficient Σ (in mm) is required to rescale the contribution of each component in order to explain the original displacement data set. Since the vbICA belongs to the field of linear decompositions, we can write the result of the decomposition of the data matrix *X* as:

(2) $$X_{M\times T}=\;U_{M\times L}\Sigma_{L\times L}V_{L\times T}^{T}$$

*U*_*M×L*_ embeds the spatial response of the *M* time‐series to the *L* sources of displacement; *V*_*L×T*_ embeds the temporal evolution of the *L* sources; Σ _*L×L*_ is a diagonal matrix containing the relative importance of the different ICs in explaining the displacement data set (in mm). The configuration is such that we look for independent signals in the time domain, that is, the sources correspond to the columns of the *V* matrix while the mixing matrix is given by the product *U*Σ. The vbICA algorithm models the probability density function of each source via a mix of Gaussian distributions (4 in this case, as suggested by Choudrey, 2002), retrieving the spatial and temporal information of the independent sources of deformation. We exclude from this analysis all stations without data after the Amatrice mainshock and stations with large data gaps (>90%) across the 2016–2017 earthquake sequence. The sufficient number of sources (*L*) is determined by performing statistical tests such as the *F*‐test (Kositsky & Avouac, 2010) and the ARD test (Choudrey, 2002; Gualandi, Avouac, et al., 2016; Gualandi, Serpelloni, & Belardinelli, 2016). We perform the decomposition with a number of ICs *L = *3, 4, 5, 6, and the ARD test limited *L* ≤ 5. We also perform the *F*‐test, which suggests to retain four components and such configuration is the one we investigate.

### 2.3 Results

**Figure 3:**
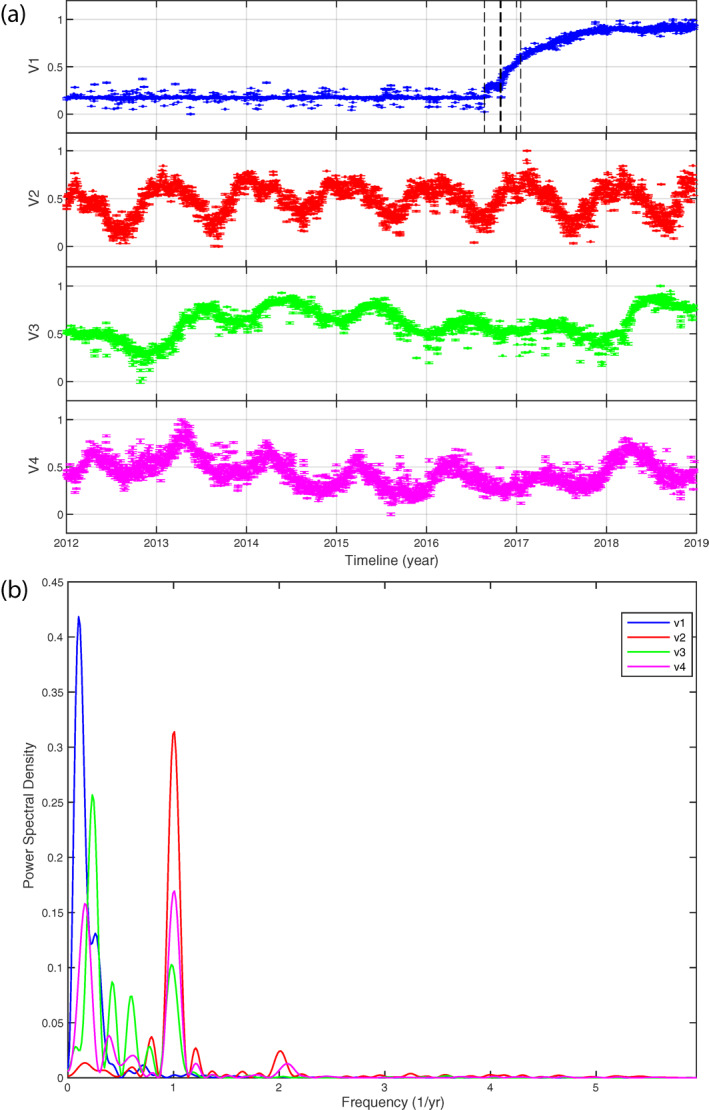
(a) The temporal evolution *V* of the four independent components (vertical dashed lines for *V*1 mark the Amatrice, the Visso‐Norcia and the January 2017 Campotosto earthquakes) and (b) their corresponding power spectral density plots.

**Figure 4:**
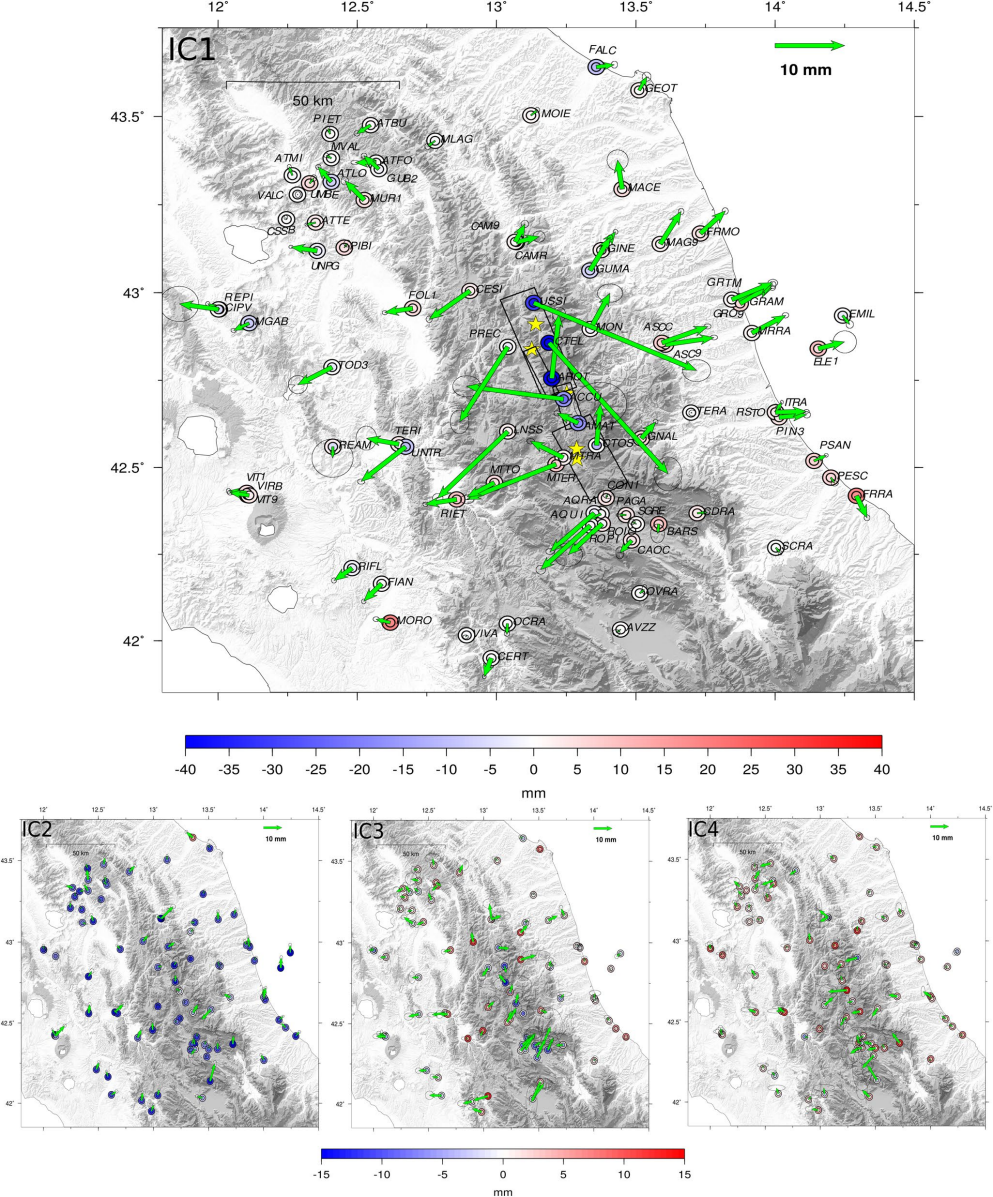
In Figure, the dimensional spatial components (Σ *U*) of the IC1, IC2, IC3, IC4, with the corresponding temporal functions being normalized between 0 and 1 (Figure 3a). Green arrows mark the horizontal response in mm; outer colored circles mark the vertical response +*σ* while inner colored circles mark the vertical response −*σ* of the Global Positioning System stations (in mm). Error ellipse shows the uncertainty associated with the ICs at 2*σ*. Yellow stars mark the location of the main events of the seismic sequence (as in Figure 1), while the black boxes in IC1 show the location of the faults responsible for the 2016–2017 sequence as in Cheloni et al. (2017) and Cheloni, Falcucci, and Gori (2019).

The temporal evolution of the four ICs (*V*) is shown in Figure 3, together with the corresponding power spectral density plots. The ICA decomposition results in: (a) a post‐seismic relaxation signal (IC1); (b) two components with annual periodicity (IC2 and IC4); (c) a multiannual component (IC3). From the spectral analysis, it is clear that low frequencies are the most significant for the post‐seismic and the multiannual IC and that the dominant periodicity is about 1 year for the annual components. However, the IC4 shows a second peak at low frequencies as well. The spatial response of the four ICs (*U*) is shown in Figure 4. From Figure 4, the NE‐SW main direction of the *U*1 spatial pattern is rather clear, consistently with the extensional mechanisms of the seismic sequence. *U*2 shows a clear vertical motion, with all stations moving coherently up and down with annual periodicity, whereas *U*3 and *U*4 show more complex horizontal, and vertical, spatial patterns. In the next section, we will provide a physical explanation for the second, third, and fourth components, whereas the first component will be discussed in Section 4.

In order to better assess the uncertainties associated with the ICs, we run a Monte Carlo simulation generating synthetic data sets resembling the original one and performing a decomposition on each synthetic data set. From the *U*, Σ, and *V* distributions, we obtain a more realistic estimate of the uncertainties associated with the decomposition, and they are generally larger than those provided by the vbICA algorithm. We detail the procedure in Section S.1.2.

We also investigate the pre‐seismic phase, limiting the analysis to the 2015–2016.64 (i.e., the 24th of August) time‐interval as in Vičič et al. (2020), in order to detect possible deformation signals that can be associated with the preparatory phase of the 2016 earthquake sequence. According to an *F*‐test, the most suitable decomposition is the one with *L* = 4 components; we carry out the decomposition on the whole GPS network (see Figure 2). The results, reported in Text S2, show that the temporal and spatial parts of the independent components retrieved in this time span do not highlight any localized deformation signal that can be associated with a clear tectonic strain transient, such as the sudden rise at the beginning of 2016 described by Vičič et al. (2020). It is worth considering that with respect to Vičič et al. (2020), we handle differently the missing data and analyzed three‐dimensional time‐series, while they described a “precursory” signal when analyzing only the east component. A more detailed analysis of the eventual pre‐seismic slip is left for future work, since here we want to focus on the modeling of the post‐seismic deformation.

## 3 Hydrological Components

In this section, we discuss the deformation signals associated with the second, third, and fourth ICs (Figures 3 and 4), providing physical explanations for these signals. In particular, we test the hypothesis that IC2, IC3, and IC4 are associated with different hydrological processes. These processes can evenly affect the entire network or be very sensitive to the geologic features of the area; furthermore, the impact on vertical and horizontal components can be variable too. Because of the multi‐annual temporal signature of IC3, separating this signal from the tectonic deformation is also important in order to improve the accuracies of the retrieved pre‐ and post‐seismic displacements, particularly in case these are expected to be rather small.

### 3.1 IC2

IC2 represents a common mode annual signal, with a uniform spatial response (i.e., all the GPS stations move toward the same direction) in the vertical component, describing uplift of the GPS network as temperature rises and subsidence when the temperature decreases. Seasonal vertical displacements are interpreted in the literature as caused by loading due to mass redistribution in the shallow Earth crust and surface (e.g., Amos et al., 2014; Argus et al., 2014; Borsa et al., 2014; Dong et al., 2002; Tregoning, 2005). IC2 describes large vertical seasonal displacements, with median amplitude of ∼6.3 mm, and smaller horizontal displacements (median amplitudes of ∼1.1 and 0.7 mm in the N‐S and E‐W direction, respectively).

**Figure 5:**
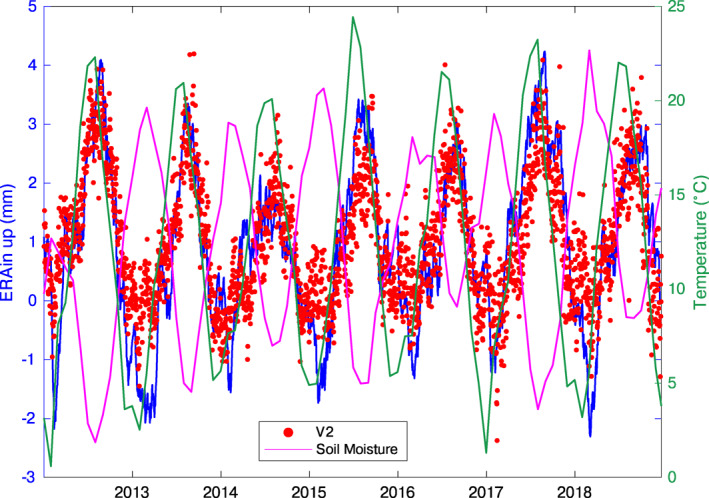
Red: V2 (sign reversed to indicate maximum uplift/subsidence during positive/negative peak values); blue: mean vertical displacements caused by surface hydrological loading using the ERA‐interim model (http://loading.u-strasbg.fr); magenta: soil moisture in the first 2 m estimated by GLDAS‐Noah (Rodell et al., 2004); green: mean monthly temperature (GHCN Gridded data provided by the NOAA/OAR/ESRL PSL, Boulder, Colorado, USA, from their website at https://psl.noaa.gov/ (Fan & van den Dool, 2008). For all the data sets, we considered mean values in a box with limits: lon. 12.00–14.50°E; lat. 42.00–44.00°N.

We compare the temporal evolution of IC2 with products of global reanalysis models estimating the redistribution of fluids at the Earth's surface. In particular, Figure 5 shows V2 compared with the temporal evolution of hydrological loading displacements estimated from the ERA‐interim (European Centre for Medium‐Range Weather Forecasts, ECMWF reanalysis) model (Berrisford et al., 2009; Dee et al., 2011), using predictions provided by http://loading.u-strasbg.fr (Gegout et al., 2010; see also Serpelloni et al., 2018 for a similar application), and with the sum of the soil moisture and the snow water equivalent in the first 2 m, estimated by GLDAS‐Noah (Rodell et al., 2004). Clearly, V2 is temporally correlated with ERA‐interim displacements and anti‐correlated with soil moisture, suggesting that IC2 is associated with surface hydrological mass loading (SHL) processes. The V2 correlation with the ERA‐interim data set is high both in terms of temporal evolution (Pearson correlation coefficient = 0.8, with no significant time lags between the two curves), and amplitude. In fact, the mean seasonal amplitude of the vertical displacements caused by surface hydrological loading according to the ERA‐interim model is 5.1 mm, which is just 1.2 mm less than the median value of the seasonal displacement associated with IC2. Concerning the horizontal displacements, the north component of the displacements associated with IC2 well correlates with predictions from the ERA‐interim model, while the east‐west IC2 displacements are weakly correlated with the ones modeled by ERA‐Interim, as already observed in Serpelloni et al. (2018). This discrepancy is likely due to limitations of assuming an elastic spherical Earth model, which does not take into account lateral heterogeneities of the Earth's elastic properties (Chanard et al., 2018).

Since the temporal evolution of IC2 is in phase with the temperature (see Figure 5), we do not exclude that monument thermal expansion may also have an effect on GPS height changes.

### 3.2 IC3 and IC4

Concerning IC3, we test the hypothesis that this multi‐annual deformation signal is associated with changes in groundwater content, as deformation associated with this process has been shown to affect the horizontal components of displacement (e.g., Serpelloni et al., 2018; Silverii et al., 2016).

**Figure 6:**
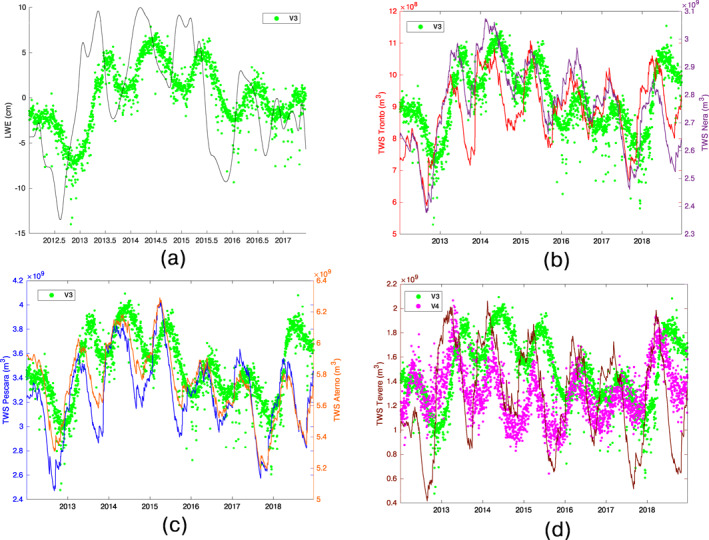
Comparison of the temporal function V3 (green dots) with: (a) liquid water equivalent thickness (gray line) from GRACE data; (b) total water storage (TWS) changes computed in the hydrological basins of Tronto (red line) and Nera (purple line) rivers; (c) same as (b) for the Pescara (blue line) and Aterno (orange line) basins; (d) TWS changes computed in the Tevere basin (brown line) and V4 (magenta dots). The extension and position of the hydrological basins are shown in Figure S3.

We use the lumped parameter hydrological model GR5J (Pushpalatha et al., 2011) to quantify daily total water storage (TWS) changes of five hydrological basins (Tevere, Nera, Tronto, Aterno, and Pescara; see Section S3). Figure 6 shows V3 and V4 compared with TWS changes estimated for the five basins and with liquid water equivalent thickness (LWE, Figure 6a) estimated by GRACE measurements processed at JPL using the Mascon approach (Version2/RL06, Watkins et al., 2015). V3 well correlates with most of the TWS estimates that anticipate the geodetic signal of about 2–3 months (Table S1). Similarly, V3 shows agreement with the temporal evolution of LWE, but since LWE data are monthly and V3 is daily it is not possible to accurately estimate neither the delay between the two signals nor the correlation coefficient. The correlation between TWS and V4 is lower and the V4‐TWS delay is less than 1 month. Pearson correlation coefficients and time lags among the aforementioned signals are reported in Tables S1 and S2.

### 3.3 Interpretation

LWE and TWS estimates do not take into account only the superficial water accumulation, as SHL models do, but also consider the effect of the deep waters. While SHL is almost spatially uniform, since it is mainly caused by the soil moisture, the accumulation of water at depth is much more heterogeneous, especially in carbonatic mountainous regions where significant groundwater flows are present.

A possible interpretation of IC2 and IC3 is that precipitation water, once removed the runoff and the evapotranspiration contributions, is partially absorbed by the first 1–2 m of soil, causing the displacements associated with IC2, which, in fact, are not significantly delayed with respect to the displacements caused by SHL. The remaining portion of precipitation may penetrate, depending on the hydro‐geological properties of the subsurface, hundreds of meters until reaching a less permeable layer, accumulating water and causing the ground displacements associated with IC3. The duration of this percolation process causes a temporal delay between TWS variations and the displacements it produced (i.e., displacements associated with IC3; see also Table S1 for the time lag values), which happen when the water level of the aquifer finally increases.

Concerning IC4, its interpretation is less straightforward. We observe that the TWS computed in the Tevere basin is the one that differs the most from the others (Figure 6): it has the lowest correlation with V3 among the basins considered (Table S1), but the highest one, when considering V4 (Table S2). Our interpretation is that IC3 alone is not sufficient to well reproduce the displacements associated with TWS changes in all the basins considered, in particular in the Tevere which include a significant number of GPS stations, so that IC4 is needed.

## 4 Post‐Seismic Relaxation

**Figure 7:**
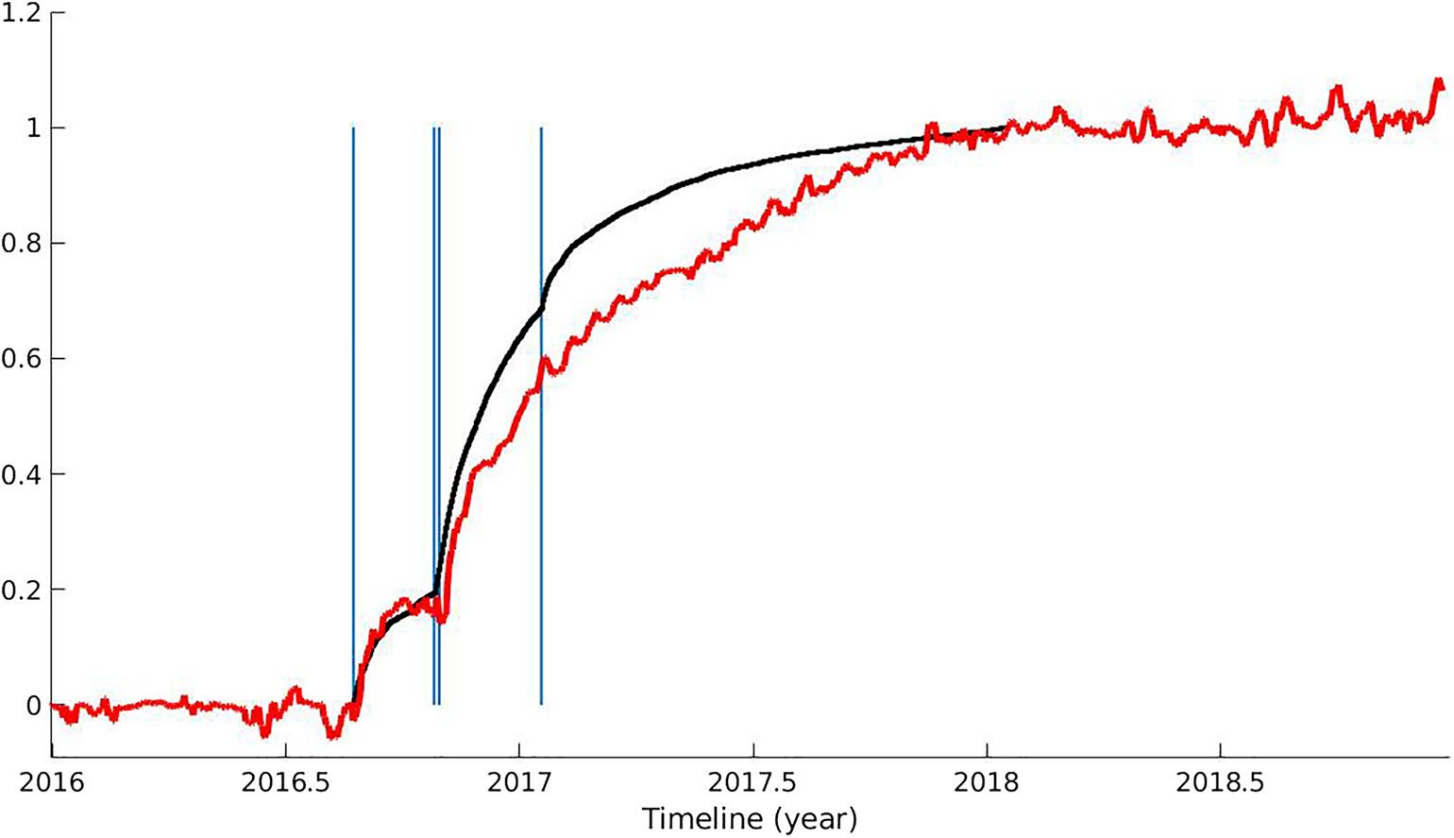
Normalized cumulative number of aftershocks from the catalog described in Michele et al. (2020) (black line) and the post‐seismic evolution represented by the temporal function V1 (red line). Vertical lines mark the epochs respectively of the Amatrice, Visso, Norcia, and the January 2017 Campotosto earthquakes.

The post‐seismic relaxation signal is mapped in the first independent component with two post‐seismic decays, the first following the Amatrice event and the second one following the Visso and Norcia events (Figures 3a and 7). Explaining the whole post‐seismic sequence with a single IC indicates a limitation of the signal separation. In fact, we would expect, from a physical point of view, at least three regions independently activated by afterslip surrounding the corresponding mainshocks distributions. We made an attempt to separate these expected relaxations performing an ICA on the time series filtered from the seasonal components retrieved in the first analysis (those discussed in Section 3), but no further ICs related to post‐seismic relaxation processes could be extracted (more details of this analysis can be found in Section S4). Therefore, in this work, we consider IC1 as representative of the whole post‐seismic deformation and we will discuss the limitations associated with this interpretation in Section 5.

### 4.1 Inversion Method

One interpretation of a post‐seismic relaxation process is represented by the occurrence of afterslip on faults. In this study, we consider as primary faults those structures already mentioned in Section 1, namely the M. Vettore fault, its antithetic fault and the Laga Mountains fault (see also Figure 1). The segmentation of this fault system is not unequivocally defined in the literature: some studies propose coseismic slip on a single plane for the seismic sequence (Cheloni, Falcucci, & Gori, 2019; Xu et al., 2017), whereas others (Chiaraluce, Di Stefano, et al., 2017; Cheloni et al., 2017; Walters et al., 2018) divide such fault plane into 3–4 segments which are consistent in terms of strike and dip. Some authors (e.g., Cheloni et al., 2017; Scognamiglio et al., 2018; Walters et al., 2018) invoke for the Norcia earthquake the activation of multiple secondary faults, in particular a fault antithetic to the M. Vettore fault well highlighted by the seismicity (Chiaraluce, Di Stefano, et al., 2017). However, more recent studies (Cheloni, Falcucci, & Gori, 2019; Pousse‐Beltran et al., 2020) suggest that the main fault system plus an antithetic fault is sufficient to explain the complex displacement pattern observed. Here we keep the faults' geometry as simple as possible, considering also the number of GPS stations available. In order to take into account the four Mw > 5 events of January 2017, we extend the model southwards to the Campotosto fault segment. The post‐seismic displacement recorded at the GPS sites in the Campotosto and Paganica area suggest a potential partial aseismic reactivation of the Paganica fault (see Text S5 for more details).

**Figure 8:**
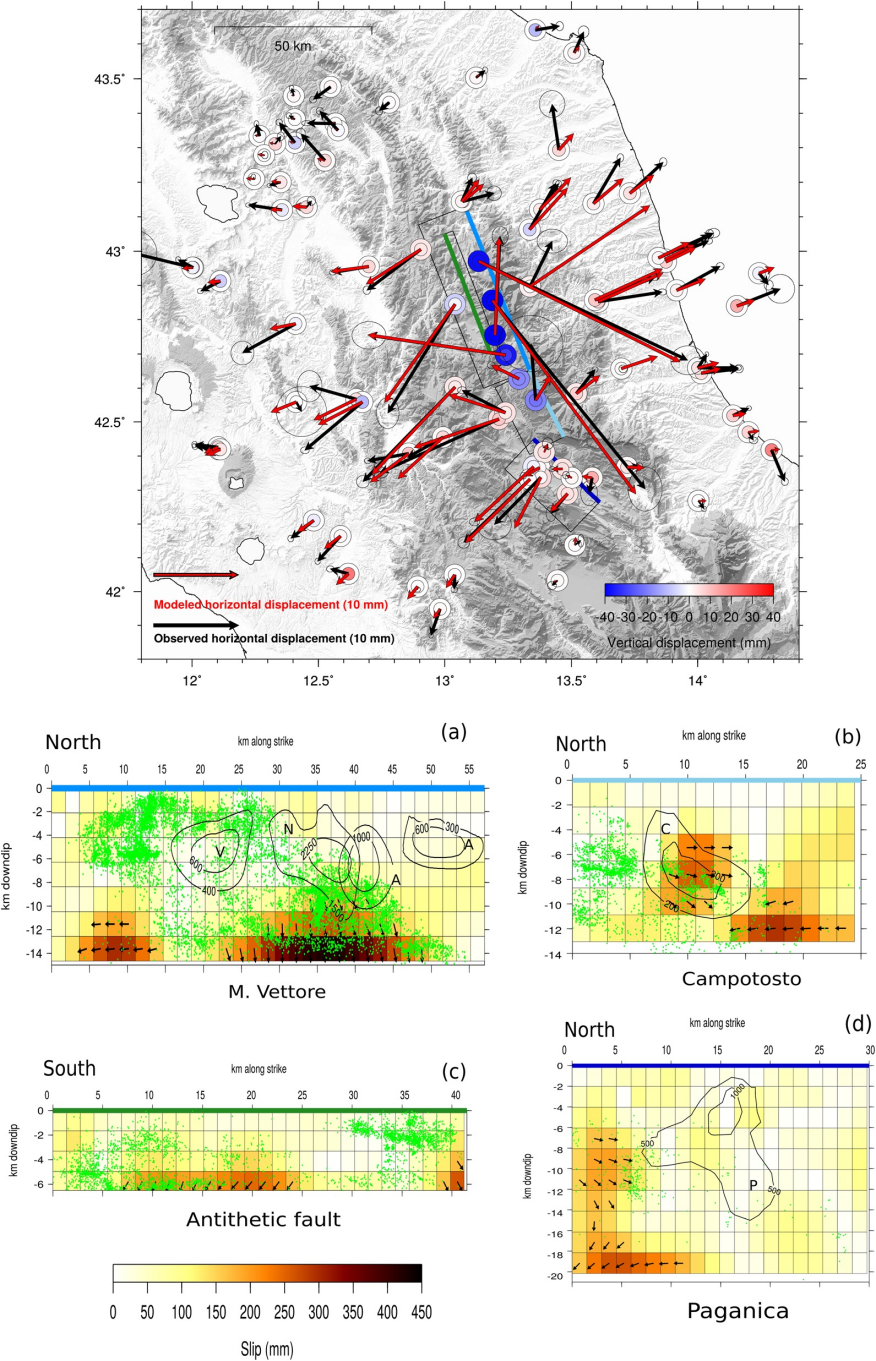
Map: black and red arrows represent respectively the observed and the modeled horizontal post‐seismic cumulative displacement on the 24th of August 2016–January 2019 time interval, whereas inner and outer circles represent respectively the observed and the modeled vertical post‐seismic cumulative displacement. Error ellipse as in Figure 4. Solid lines show the surface projection of the high angle faults described in Section 4.1. The faults' traces are colored as in panels (a–d) which show the afterslip distribution on the M. Vettore, Campotosto, antithetic, and Paganica faults, respectively, in a strike‐dip reference system (slip in mm). Co‐seismic contours on the M. Vettore fault are from Cheloni et al. (2017) and Cheloni, Falcucci, and Gori (2019) (V, N, A are respectively for Visso, Norcia, and Amatrice earthquakes), on the Campotosto fault from Cheloni, D'Agostino, et al. (2019) (C = January 2017 earthquakes), on the Paganica fault are from Gualandi et al. (2014) (P = L'Aquila 2009 earthquake).

We adopt the dip angles of the co‐seismic studies Cheloni et al. (2017), Cheloni, Falcucci, and Gori (2019), and Walters et al. (2018). The northern master fault here considered, resembles the M. Vettore fault from Cheloni, Falcucci, and Gori (2019) but it is furtherly extended along the strike direction as suggested by the presence of seismicity, and from now on, we will refer to it simply as M. Vettore fault. For similar reasons, we consider a fault antithetical to the M. Vettore fault, for which we adopt the same dip and strike angle as in Cheloni et al. (2017), Cheloni, Falcucci, and Gori (2019), and Walters et al. (2018), but is extended northwards. The southern master fault here considered unites part of the Gorzano fault in the Cheloni et al. (2017)'s notation (the Laga fault in Walters et al., 2018's notation) and the Campotosto fault from Gualandi et al. (2014) and will be simply referred to as Campotosto fault. The Paganica fault is included following the Gualandi et al. (2014)'s geometry. Recent studies (e.g., Michele et al., 2020; Vuan et al., 2017) agree that the down‐dip extension of the faults activated during the seismic sequence is bounded at depth by a subhorizontal thick layer of seismicity (Section 1) at a depth of about 10 km, which is consistent with the thickness of the brittle crust estimated by Boncio et al. (2004) for this area. All the faults are discretized in sub‐faults of about 2 × 2 km^2^ (Figure 8).

For the slip inversion, we follow the conceptual scheme proposed by Kositsky and Avouac (2010) and adapted to the ICA decomposition by Gualandi, Avouac, et al. (2016) and Gualandi, Serpelloni, and Belardinelli (2016). In practice, we invert the spatial pattern relative to the post‐seismic IC and then we recombine the retrieved spatial slip distribution with the corresponding weight Σ and temporal function *V*. The linear system we are dealing with is described by the relation

(3) $$d=U_{1}=Gm$$

where the data vector *d* is the spatial deformation associated with the IC1, *G* stands for the Green's functions for the fault system, *m* = (*m*_strike_, *m*_dip_) is the afterslip spatial distribution along the strike and the dip directions. The inversion follows the least squares formulation of Tarantola (2005) for linear problems:

(4) $$m=m_{0}+C_{m_{0}}G^{T}{\left(GC_{m_{0}}G^{T}+C_{d}\right)}^{-1}\left(d-Gm_{\text{0}}\right)$$

(5) $$C_{m}=C_{m_{0}}-C_{m_{0}}G^{T}{\left(GC_{m_{0}}G^{T}+C_{d}\right)}^{-1}GC_{m_{0}}$$

where *m*_0_ and $C_{m_{0}}$ represent respectively the a priori model and its covariance matrix, *G* are the Green's functions for a homogeneous elastic half‐space, *d* is the data vector, and *C*_*d*_ the corresponding covariance matrix which embeds the uncertainties relative to the spatial part of the inverted IC (i.e., *U*1) calculated with the procedure described in Section 2.3. We impose an additional positivity constraint to account for the dip‐slip tectonic setting (i.e., *m*_dip_ ≤ 0). We follow the formalism of Radiguet et al. (2011), using a null a priori model and considering the spatial correlation of slip on patches to decay exponentially. Provided that *A* and *B* are two fault patches at a distance *d*_*AB*, the element *AB* of the prior covariance matrix can be written as:

(6) $$C_{m_{0}}^{AB}={\left(\sigma_{m}\lambda_{0}/\lambda \right)}^{2}e^{-d\_AB/\lambda }$$

where *λ* is the characteristic decay length, *λ*_0_ is a scaling length factor fixed to the root square of the average of the patches' area, *σ*_*m*_ is a standard deviation of the a priori model parameters. The inversion needs to be regularized determining the values (*λ*, *σ*_*m*_). The regularization parameters (*λ*, *σ*_*m*_) associated with each fault depend on the dimension of the fault itself, therefore a unique set of values cannot be selected for the whole fault system. They are fixed seeking the best compromise between a physically acceptable solution (i.e., compatible with the tectonic setting) and the misfit with the data, and they resulted in a *λ* = 2 × *λ*_0_ and a priori standard deviation *σ*_*m*_ = 0.71 for the M. Vettore and its antithetic fault and *σ*_*m*_ = 1 for the Campotosto and the Paganica faults.

### 4.2 Inversion Results

The resulting afterslip distribution solution is shown in Figure 8: it satisfactorily reproduces the data, with an almost perfect reconstruction of the displacement pattern in the epicentral area, whereas more distant GPS sites show a weaker agreement (Figure 8).

This model shows the occurrence of slip on the deepest portion of the M. Vettore fault below the co‐seismic ruptures of the Amatrice and Norcia earthquakes, with a maximum slip >40 cm and a prevalent normal mechanism (Figure 8a), while below the Visso area transcurrent slip reaches up to ∼25–30 cm (both slipping areas being at ∼9.5 km depth). Contextually the antithetic fault, activated by the Mw 5.4 event that occurred 1 h after the Amatrice main event (Chiaraluce, Di Stefano, et al., 2017), accommodates normal slip (∼25 cm) in its deepest part (∼5.5 km depth), where the fault meets the M. Vettore main fault. The Campotosto fault accommodates some slip about 10 km south of the town of Amatrice with a maximum slip of ∼25 cm (∼6 km depth) and, with a similar intensity, about 10 km southwards (∼10 km depth). Our solution suggests the presence of aseismic slip on the northernmost edge of the Paganica fault, outside the 2009 L'Aquila earthquake co‐seismic ruptures as shown in Figure 8d (Gualandi et al., 2014; Ragon et al., 2019).

As it can be observed from Figure 8, the afterslip mechanism is not sufficient to explain the ∼2.3 years cumulative displacement recorded by the whole GPS network. Remarkably, the displacement produced at sites farther from the epicentral area during the post‐seismic phase appears to have a signal‐to‐noise ratio >1 (see error ellipse in Figure 4), however they are generally underestimated by the modeled afterslip distribution. In order to better highlight this fact, we compare the elastic response of the GPS stations to a homogeneous slip of 1 m on a 60 km long, 10 km deep rectangular fault plane, which represents an along‐strike extension of the major structures described in Cheloni et al. (2017) and Cheloni, Falcucci, and Gori (2019), with the normalized L2 norm relative to the IC1 spatial response at the studied stations (a more detailed description can be found in Text S6). This procedure allows us to identify those sites (red triangles in Figure S10a) that cannot be modeled only through slip on the major structures involved in the seismic sequence (“far‐field” sites): indeed we notice from Figure S10b that the elastic afterslip model systematically underestimates the IC1 displacement at far‐field sites, suggesting that a purely elastic mechanism is not sufficient to justify the displacement records of the whole data set. As Figure 8 shows, this fact remains true in spite of the strong concentration of afterslip on the deepest patches of the high angle faults.

### 4.3 Afterslip on the Shear Zone

**Figure 9:**
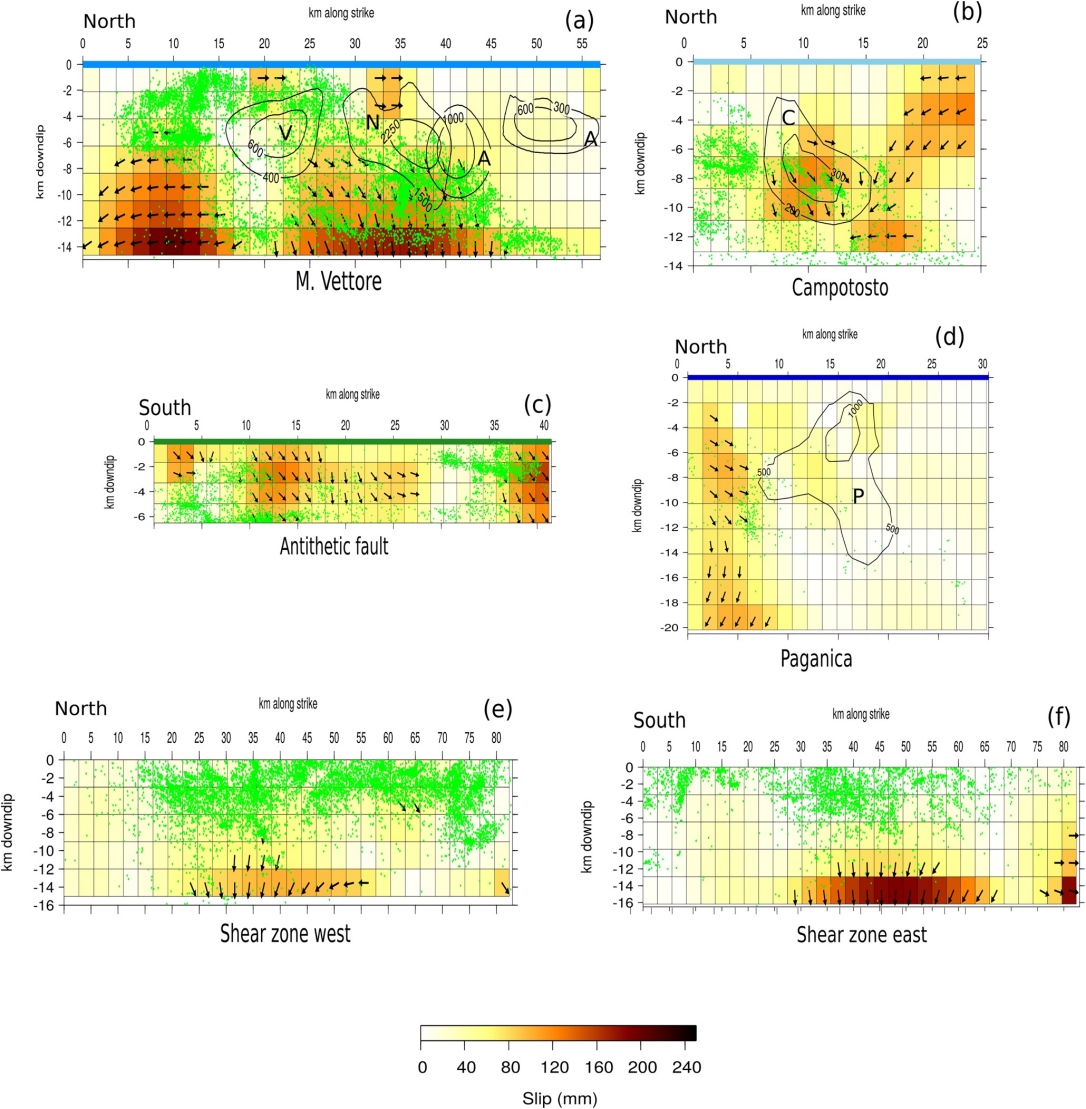
Panels (a–d) show the afterslip distribution respectively on the M. Vettore, Campotosto, antithetic, and Paganica faults in a strike‐dip reference system. Panels (e and f) show the afterslip distribution on the western and eastern segments of the shear zone (slip in mm). Co‐seismic contours on the M. Vettore fault are from Cheloni et al. (2017) and Cheloni, Falcucci, and Gori (2019) (V, N, A are respectively for Visso, Norcia, and Amatrice earthquakes), on the Campotosto fault from Cheloni, D'Agostino, et al. (2019) (C = January 2017 earthquakes), on the Paganica fault are from Gualandi et al. (2014) (P = L'Aquila 2009 earthquake).

**Figure 10:**
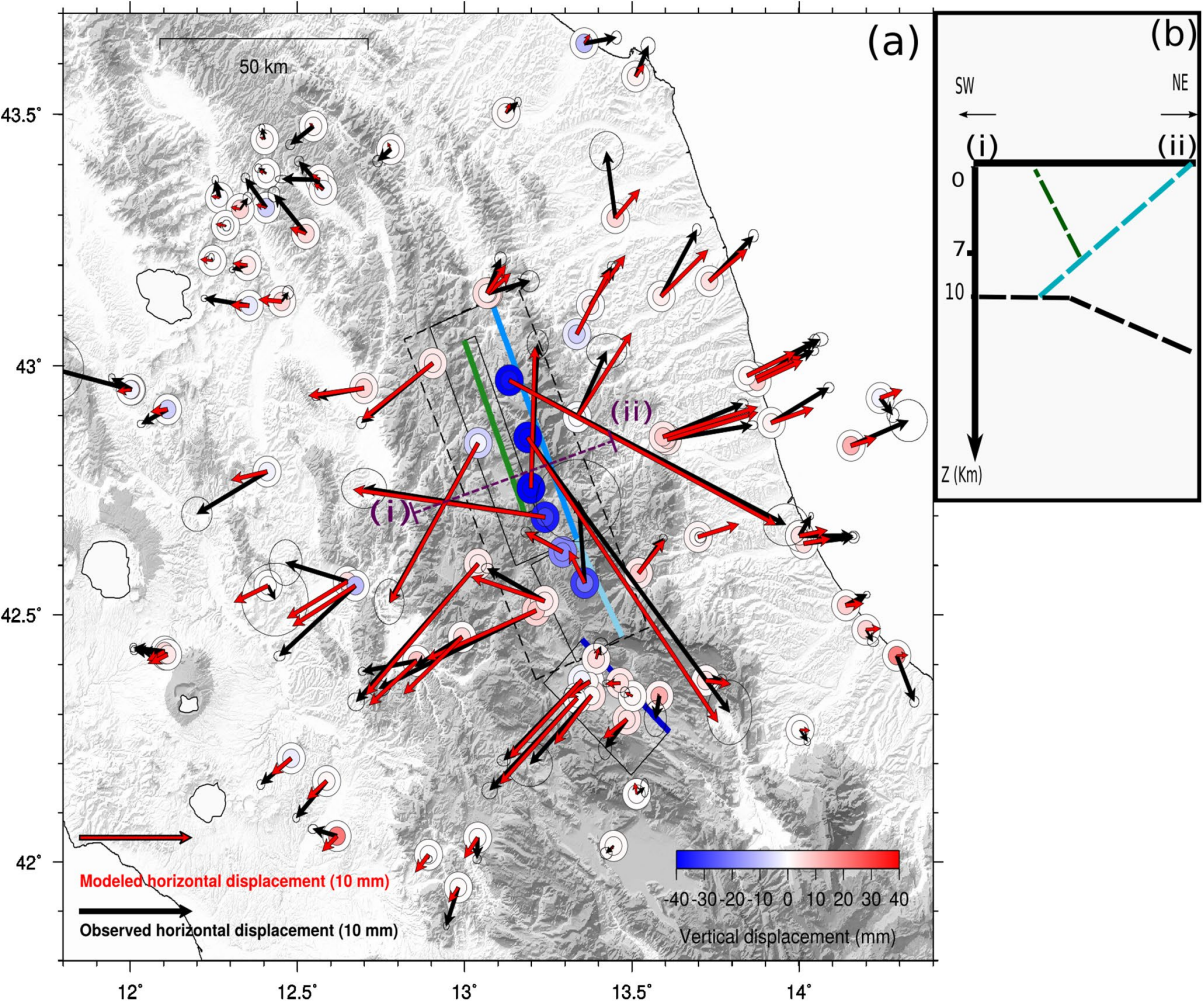
(a) Black and red arrows represent respectively the observed and the modeled horizontal post‐seismic cumulative displacement, whereas inner and outer circles represent respectively the observed and the modeled vertical post‐seismic cumulative displacement. Error ellipse as in Figure 4. Solid lines show the surface projection of the high faults described in Section 4.1 and dashed lines the surface projection of the shear zone as described in Section 4.3. The faults' traces are colored as in panels (a–d) of Figure 9. (b) We show a cross section of the fault system along the (i–ii) line.

As already mentioned in Section 1, many authors (e.g., Chiaraluce, Di Stefano, et al., 2017; Michele et al., 2020) pointed out, at about 10 km depth, the presence of the shear zone, namely a ∼2–3‐km‐thick subhorizontal layer of seismicity. Afterslip in the shear zone has been proposed by Vuan et al. (2017), and Pousse‐Beltran et al. (2020) infer a possible contribution of the shear zone to the displacement field, but do not include it in their formal analysis. To further investigate its potential role during the post‐seismic phase, we carry out the inversion of our data set following the same procedure described in Section 4.1, including the shear zone as two planar surfaces. We model it according to the interpretations shown in Figure 10b of Michele et al. (2020) and in Figure 1 of Vuan et al. (2017), with a low‐angle east dipping plane and an almost flat detachment west of it. The two planes are discretized into sub‐faults of about 3 × 3 km^2^ (Figures 9e, 9f and 10b).

When we include the shear zone in the inversion of the data, slip on the four high‐angle faults (Figure 8) is not concentrated on the deeper patches only but it occurs on shallower patches as well, mainly bordering the areas already co‐seismically activated. This is true in particular for the M. Vettore and its antithetic fault (Figures 9a and 9c), where afterslip is more distributed and its maximum intensity is reduced (max of slip ∼20 cm). On the other hand, on the southern structures (i.e., the Campotosto and Paganica faults, Figures 9b and 9d), we observe a reduction in the amount of slip, but the inclusion of the shear zone does not substantially change the areas involved in the post‐seismic phase (for a comparison between the two inversion models see Figure S16).

Moreover the western, flat, fault undergoes slip in an area that would correspond to the down‐dip extension of the M. Vettore fault slipping area, reaching the maximum (∼10 cm) on its deepest edge (Figure 9e). The eastern part of the shear zone (Figure 9f) shows a concentration of afterslip on the deepest patches as well but with greater intensities (∼18–20 cm). For what concerns the data reconstruction (Figure 10a), we find a slightly better reproduction of the displacement for some near field sites, with respect to the model of Section 4.2 (Figure S17). Concerning the far‐field stations, on the Adriatic side, we find an improvement in the fit (WRMSE improvement ∼16.5%, see Table S3) whereas for the Tyrrhenian side this model only marginally improves the fit at far‐field stations (WRMSE improvement ∼0.5%, Table S3). While it is true that the fit to the data on the whole data set is generally improved (WRMSE improvement ∼6%, Table S3), the resolution matrix is overall low (Figure S18), with values larger than 0.2 only close to the GPS stations, as expected. The restitution matrix shows instead results larger than 1 on the majority of the patches (Figure S19). This result suggests that even with a complicated model like this, further slip is asked by the data. Bearing in mind the structural constraints imposed on the fault geometry by previous studies, we think that this result points toward the need to invoke also a viscoelastic relaxation mechanism to explain the geodetic displacements.

### 4.4 Viscoelastic Relaxation

From Figure 10a, we notice a general underestimate of the displacement pattern recorded at far‐field GPS sites with much of the afterslip localized in the deepest portions of the faults. As it is suggested by Riva et al. (2007) for the Colfiorito seismic sequence, afterslip at the base of the seismogenic layer might reflect a rheological discontinuity between the upper brittle crust and the underlying layers, herein referred to as the brittle‐ductile transition. Furthermore, as many authors (e.g., Perfettini & Avouac, 2007) showed, a correlation in time between afterslip and the cumulative number of aftershocks exists. In this case, it remains true up to a few months after the Amatrice earthquake only (Figure 7), corroborating the hypothesis of other post‐seismic mechanisms acting after the Norcia mainshock besides afterslip.

**Figure 11:**
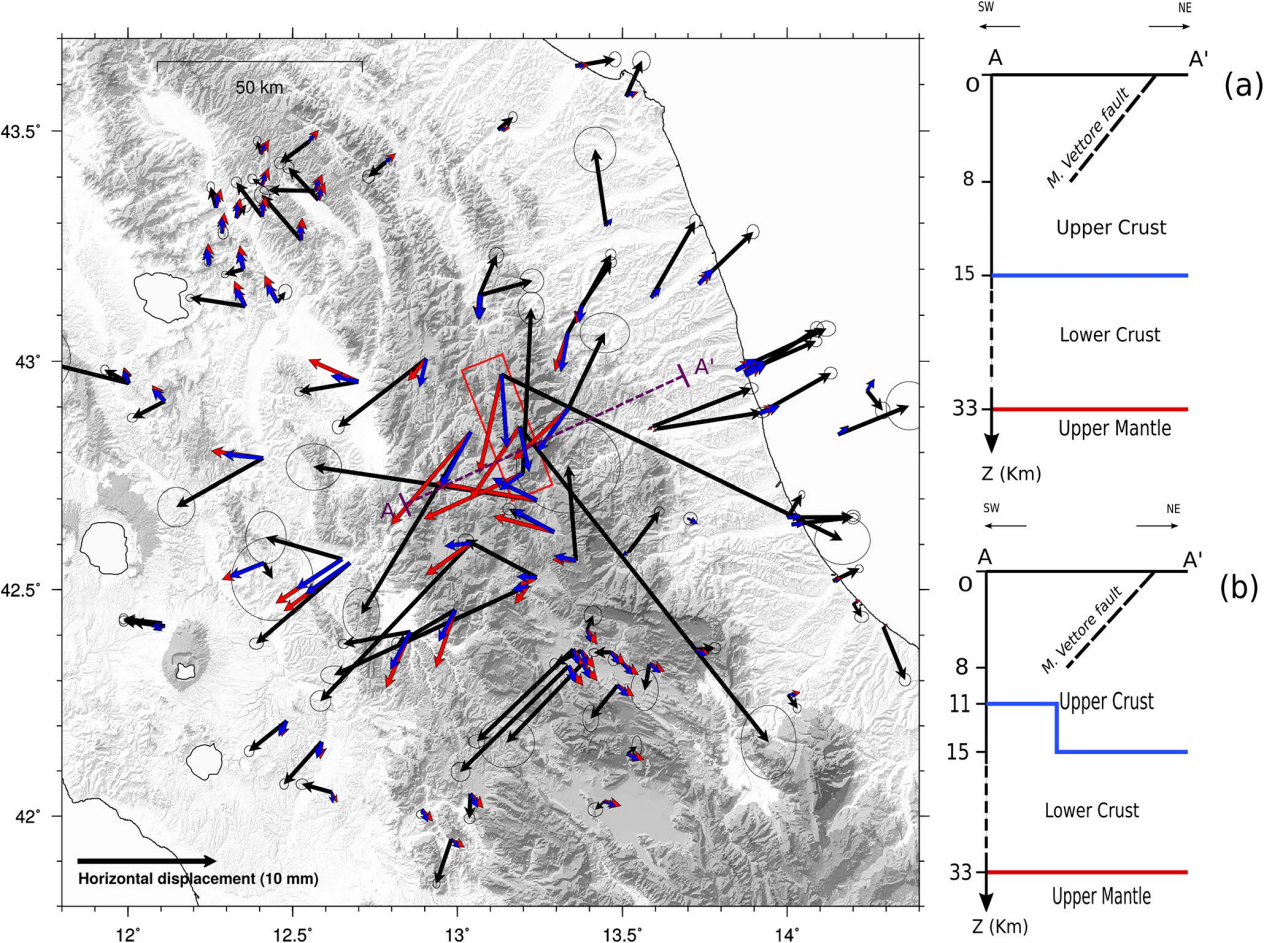
In map, we compare the post‐seismic IC (black arrows) and the viscoelastic relaxation field after 2.1 years from the 30th of October Norcia mainshock (blue arrows for the model with a constant brittle‐ductile transition's depth, red arrows for the model with a variable transition depth). Error ellipses as in Figure 4. The red box marks the surface projection of the (Cheloni, Falcucci, & Gori, 2019)'s masterfault. (a and b) Cross sections along the AA' line respectively for the model with constant and variable brittle‐ductile transition's depth.

We thus investigate a possible contribution of the viscoelastic lower crust and upper mantle to the displacement field. We use the open‐source software RELAX 1.0.7 (Barbot & Fialko, 2010). For simplicity, we consider here as an initial stress perturbation the one generated by the coseismic slip distribution of the major event of the sequence (i.e., the Norcia Mw 6.5 earthquake) as described in Cheloni, Falcucci, and Gori (2019). We model the viscoelastic medium through Maxwell rheologies even though we are aware of the potential importance of power‐law rheologies in controlling viscoelastic relaxation (e.g., Freed & Burgmann, 2004). Due to the length of our post‐seismic time series (∼2 years) and the relaxation rates in the order of a few millimeters per year characteristics of moderate earthquakes (Riva et al., 2007), it is possible that our data cannot properly distinguish the differences with other rheologies. The rheological profile we implement (Figure 11a) consists of a brittle upper crust (elastic parameters *λ* = *μ* = 30 GPa) that overlays the lower crust and the lithospheric mantle located below 33 km depth (Pousse‐Beltran et al., 2020).

As already acknowledged by Freed et al. (2006), in the use of stress‐driven models to study surface deformations from viscoelastic relaxation, a key challenge is to reproduce the far‐field observations without overshooting the near‐field data: too low viscosities and/or too thick viscoelastic layers can easily reproduce the far‐field data but they greatly overestimate the near‐field data (and vice versa). The tests we performed suggest viscosity values in the range of 10^18^ < *η*_lc_ < 10^19^ Pa s for the lower crust, and a thickness of about 18 km. Taking *η*_lc_ < 10^18^ Pa s, we observe a non‐monotonic temporal evolution of the displacement at some GPS sites (Text S7) which differs from IC1. Moreover, due to the short relaxation time (in the order of 0.1 year), we predict a cm‐scale post seismic displacement, which is not justified by our data. Hence, we fix the viscosity of the lower crust *η*_lc_ = 5 × 10^18^ Pa s, and the viscosity of the mantle *η*_m_ = 10^21^ Pa s. The mantle is located too deep and its viscosity is too high for the effects of its relaxation to be observable on our data set (Riva et al., 2007). However, the same data set suggests to bound the horizontal length scale of the displacement pattern by limiting to the lower crust only the depth extent of the low viscosity region. As already observed by Freed et al. (2006) and by Hetland and Zhang (2014), vertical polarities can help to constrain the parameters of the model, namely the thickness and the viscosity of the viscoelastic layer: a thicker low viscosity layer produces a broader subsidence area that is not confirmed by our data.

**Figure 12:**
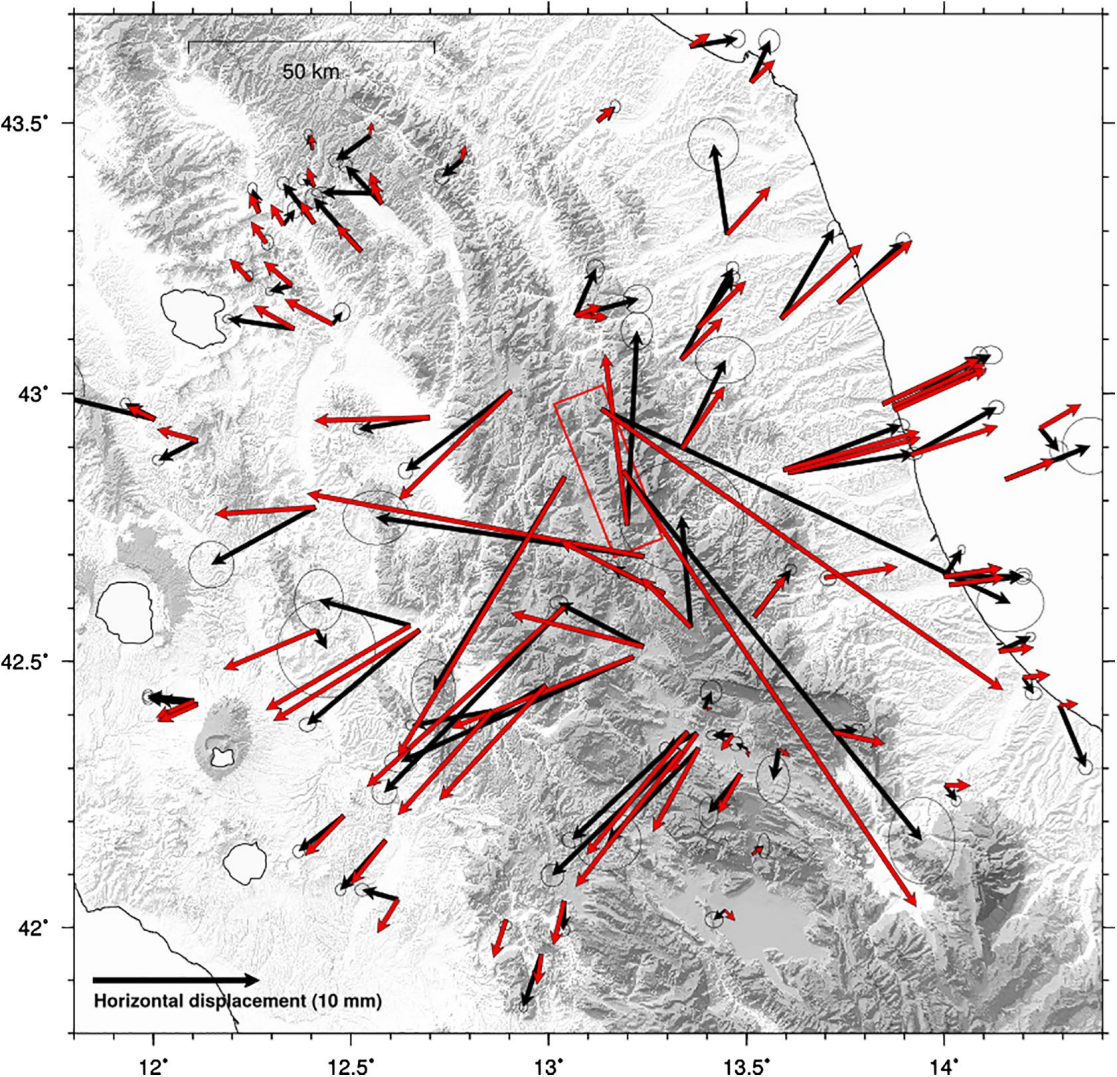
Map showing the sum (red arrows) of the displacements associated with afterslip (from model shown in Figure 9) and the displacements associated with the viscoelastic contribution obtained with uniform depth of the brittle‐ductile transition. Black arrows as in Figure 11. Error ellipses as in Figure 4.

However, in this sector of the central Apennines, several seismological studies (e.g., Di Stefano et al., 2009; Molinari & Morelli, 2011; Molinari et al., 2015; Tesauro et al., 2008) highlight a heterogeneity of the crust thicknesses showing a positive gradient for the depth of the lower crust starting from the western Tyrrhenian side up to the eastern Adriatic side, which is consistent with the eastward deepening of the brittle‐ductile transition proposed by Carminati et al. (2001), Carannante et al. (2013), Vuan et al. (2017), and modeled by Albano et al. (2020). To simulate this feature, we consider a second model in which the upper crust has a depth of 11 km on the western side of the Apennines, and increases to 15 km toward the Adriatic Sea, affecting the entire eastern side of the study area (Figure 11b). The map in Figure 11 shows the horizontal displacement field derived from the relaxation of the viscoelastic lower crust, for a variable (red arrows) and constant (blue arrows) brittle‐ductile transition depth. The ∼2 years long cumulative vertical displacement resulting from the models (∼3 mm) is below the threshold of detection of GPS for all the sites away from the epicentral area and it is therefore not represented in Figure 11. On the Adriatic Side, the two models are equivalent as well as the displacements they produce in the far‐field, which agree in direction with IC1. West of the M. Vettore fault the constant‐thickness model provides smaller displacements both in near and far field, coherently with a thicker brittle crust. According to these results, the model with uniform thicknesses (Figure 11a) allows us to find the best compromise between the near‐field and the far‐field data. Having the viscosity of the lower crust fixed, a thicker upper crust (Figure 11a) allows us to reduce the effects of the viscoelastic relaxation in the near‐field. Hence, we can explain the missing displacement at far‐field sites while not overestimating near‐field observations, which are already well modeled by afterslip (see Figure 10). The linear summation of the afterslip solution shown in Figure 9 and of the viscoelastic model with a constant brittle‐ductile transition depth is shown in Figure 12. Although a fully coupled afterslip‐viscoelastic inversion would give more accurate results, it can be observed that the very simple forward model in Figure 11a accounts for a relevant portion of the total afterslip + viscoelastic displacement at far‐field sites (see Section 4.2), with a mean value of the ratio of displacement modeled through viscoelastic relaxation and total modeled displacement = 22%.

## 5 Discussion

The analysis of the 2012–2019 time‐span GPS displacement time series allows us to highlight one post‐seismic component (IC1, Figure 3) plus three hydrological, seasonal, components (IC2, 3, 4; Figure 3). The presence of a spatially uniform vertical displacement signal for the IC2 (Figure 4), associated with the superficial hydrological loading, is consistent with other results, suggesting this usually is the largest source of non‐tectonic seasonal deformation (Michel et al., 2018; Serpelloni et al., 2018). The other two hydrological components (IC3 and IC4) are interpreted as displacements caused by underground water content variations, which can provoke mm‐scale horizontal displacements as already observed by Silverii et al. (2016) and Devoti et al. (2018) in the Apennines, and by Devoti et al. (2015), Serpelloni et al. (2018), and Pintori et al. (2021) in the Southern Alps. The seasonal displacements associated with the ICs discussed in Section 3 were not detected by the analysis of the InSAR data (Pousse‐Bertrand et al., 2020) during the pre‐seismic phase. An additional analysis performed on the pre‐seismic time‐span (Section 2.3) does not show components that can be clearly associated with transient tectonic deformations. In particular, we do not find evidence of a preparatory phase prior to the Amatrice mainshock (Text S2) similar to the one described in Vičič et al. (2020).

The separation of non‐tectonic sources of deformation is an important task, especially when dealing with mm‐scale post‐seismic displacements. This proved to be very important, for example, for the stations located in the Paganica area: such GPS sites, in fact, appear to be highly influenced by the multi‐annual hydrological component (IC3), which shows a prevalent NE‐SW horizontal deformation signature, consistent with the direction of the tectonic deformation in this area (Figure 4). The vbICA well separates the tectonic and non‐tectonic signals, and neglecting a post‐seismic contribution to the displacement of these sites leads to a biased representation of the time series for the time period after the Norcia mainshock (see Figure S7). Excluding the Paganica fault in the inversion for afterslip (see Text S5 and Figure S9a) implies a strong concentration of slip on the southern edge of the Campotosto fault (i.e., as close as possible to the GPS sites location), which seems unlikely, considering the difference in magnitude between the events nucleated on the Campotosto fault and those nucleated on the M. Vettore fault. Furthermore, slip on the Campotosto fault alone underestimates the displacement measured at the GPS sites in the Paganica area (Figure S9b). On the Paganica fault, which was responsible for the 2009 L'Aquila earthquake, no mainshock of the 2016–2017 seismic sequence occurred, but GPS displacements in the area suggest that this fault accommodated a few cm of slip (Figure 9d) between the end of 2016 and 2019. Calculating the stress perturbation in terms of Coulomb Failure Function variation (DCFF), due to the main events of the sequence, as modeled by Cheloni et al. (2017), Cheloni, Falcucci, and Gori (2019), and Cheloni, D'Agastino, et al. (2019) on the six fault planes considered for the afterslip model (Figure S20), we obtain positive values on the Paganica fault (DCFF∼0.02–0.05 MPa) favoring slip on this structure. This finding highlights how fault interactions need to be taken into account if we want to attempt a deterministic modelization of the seismic cycle.

As mentioned in Section 4, the temporal evolution of post‐seismic deformation over the studied time‐span is described by a single IC. Following Michel et al. (2018), we perform an iterative application of the ICA algorithm (see Text S4) that should enhance the accuracy of the extraction of tectonic ICs but, in our case, it does not lead to the separation of additional post‐seismic components. The fact that the post‐seismic relaxation is described solely by IC1 brings along some limitations:

(i) In terms of afterslip, since the 2016–2017 seismic sequence was characterized by several mainshocks, describing their post‐seismic phase only through the IC1 means that we cannot get any insight into the possible spatial migration or different activation times of the different faults involved in the seismic sequence. Our analysis is limited to a stationary spatial response of the slip, not allowing us to determine when the different parts of the faults actually began to slip.
(ii) Three mechanisms primarily drive the post‐seismic relaxation of stress: afterslip, poroelastic rebound, and viscoelastic flow. In this study, we considered at least two of them (afterslip and viscoelastic flow) to be active, but having a single IC representing the post‐seismic response does not allow us to account for them separately.

Poroelastic rebound effects on surface displacement are usually confined in near field regions where the afterslip contribution to the post‐seismic deformation is also dominant (e.g., Nespoli et al., 2018). For this sequence, Tung and Masterlark (2018) suggested poroelastic effects on seismicity and preliminary computations made by Pousse‐Bertrand et al. (2020) indicate that poroelastic rebound provides marginal contribution to the observed surface displacements. This is confirmed by our results, since the ICs associated with hydrologic processes do not show any anomaly after the seismic events and the GPS stations mostly affected by IC3 and IC4 (which could be partially related to fluid migration) are in the Paganica fault area, far from the epicenters. Furthermore, the density of the GPS network does not allow to identify and limit an aquifer generating poroelastic deformations.

The simultaneous action of afterslip and viscoelasticity is also supported by the different decay patterns describing the cumulative number of aftershocks and the temporal evolution of the post‐seismic deformation signal (Figure 7). The reason why the ICA does not separate such mechanisms can be due to the short time span here considered, and longer time‐series will help to address this problem in the future (e.g., Gualandi et al., 2020). As a consequence, we cannot exactly establish the relative contribution of the various deformation mechanisms to the total measured displacement field.

### 5.1 Afterslip Remarks

The afterslip distribution obtained with the four high angle faults configuration (Figure 8) results in a high concentration of afterslip at the base of the structures that, however, is not sufficient to explain the displacements observed at sites far from the epicentral area (red triangles in Figure S10a) as we show in Section 4.2. The fact that the displacement measured at such far‐field sites is statistically significant (as discussed in Section 2.3) drives our choice to add complexities to the initial model (Figure 8). First we attempt to investigate a contribution of the sub‐horizontal shear zone highlighted by seismicity, as suggested by Vuan et al. (2017) and Pousse‐Beltran et al. (2020). The inclusion of the shear zone in the inversion leads to a better fit of the far‐field GPS stations, especially on the Adriatic coast, as well as to a different slip distribution. In particular, we find a reduction of the afterslip at the bottom edges of the high angle faults (Section S8) that is likely compensated by the slip accommodated at the bottom edge of the shear zone itself (Figures 9e and 9f).

We can compare Figure 9 model with results obtained by Pousse‐Beltran et al. (2020). The latter, using InSAR measurements integrated by near‐field GPS stations, modeled slip on the M. Vettore and its antithetic fault with a geometry similar to that of Cheloni, Falcucci, and Gori (2019). Pousse‐Beltran et al. (2020) found two main slipping areas: one beneath Arquata del Tronto and one in the Castelluccio area. This latter is less certain, as claimed by the authors themselves, and it partially overlaps with the Amatrice and Norcia coseismic ruptures. The solution shown in Figure 9 suggests significant shallow slip about 10 km NE of the shallow slipping area below Arquata found by Pousse‐Beltran et al. (2020), and it does not show the overlapping with the coseismic ruptures on the M. Vettore fault (Figure 9a). Moreover the maximum amount of slip that Pousse‐Beltran et al. (2020) retrieve (∼16 cm) is less than what we have observed in our study (∼24 cm). This can partly be explained by the shorter time span considered by Pousse‐Beltran et al. (2020) (i.e., from November 1, 2016 to February 11, 2017), by the fact that the GPS data set we employ covers a much wider area than the interferograms used in Pousse‐Beltran et al. (2020), and by the smoothing method adopted during the inversion.

Figure 9b shows that the northern slipping area on the Campotosto fault overlaps with the co‐seismic slip distribution of Cheloni, D'Agostino, et al. (2019). However, their solution is derived from InSAR data that encompassed the displacement of the first month after the January 2017 mainshocks. We estimate that in that time period (i.e., up to February 11) about 8% of the moment released is due to post‐seismic relaxation. For what concerns the shear zone, it accommodates a few cm of slip on the eastern, slightly E‐dipping, side (Figure 9f). Such concentration of slip far from the co‐seismic area is likely driven in the inversion by displacements measured at sites toward the Adriatic coast. If, on the one hand, our data can hardly resolve the deeper patches of our structures (Figure S18), on the other hand, the GPS positions with respect to the faults demand deep slip to occur. The stress perturbation due to the mainshocks of the 2016–2017 sequence on those deep patches is slightly positive (DCFF ∼ 0.05–0.1 MPa, Figure S20), therefore, in principle, slip in that area is not forbidden.

The equivalent seismic moment associated with afterslip (up to January 2018) is *M*_0_^geodetic^ = 6.25 × 10^18^ Nm (for a rigidity modulus = 30 GPa) that corresponds to a Mw 6.5. In order to compare this with the seismic moment released by aftershocks, we consider the seismic catalog described by Michele et al. (2020) and convert the reported ML into Mw using the relation Mw‐ML proposed by Munafò et al. (2016) for small events. The moment released by aftershocks up to January 2018 is *M*_0_^aftershocks^ = 4.60 × 10^17^ Nm, meaning that the post‐seismic deformation was dominated by aseismic motion. Aftershocks on the Campotosto and antithetic faults overlap only partially with the patches undergoing post‐seismic slip (Figures 9b and 9c). On the M. Vettore fault (Figure 9a), a first cluster of aftershocks is located on the bottom edge of the Amatrice and Norcia co‐seismic ruptures where a large amount of afterslip is accommodated; whereas a second cluster is located in the shallower portion of the fault around the Visso slipping area. The majority of the aftershocks of this second cluster occurs outside the patches undergoing afterslip, which might be due to a lack of GPS coverage in that area.

### 5.2 Viscoelastic Remarks

The maximum value of afterslip of the inverse solution shown in Figure 9 is located at the base of the high‐angle faults, and, following Riva et al. (2007) who studied the 1997 Umbria‐Marche seismic sequence, this might be symptomatic of a rheological discontinuity decoupling the seismogenic upper crust from a viscoelastic lower crust. According to Boncio et al. (2004) beneath this sector of the Apennines, the active faults in the seismogenic layer detach into a layer dominated by aseismic plastic flow passing through a broad transition zone. Such detachment is illuminated by a high seismicity rate discussed by Vuan et al. (2017) and, following Chiaraluce, Barchi, et al. (2017), interpreted as the top of the brittle‐ductile transition. Flat detachments can mark the presence of the brittle‐ductile transition within the crust (Fayon et al., 2000; Jolivet et al., 2010; Platt et al., 2015; Rabillard et al., 2018). According to Nespoli et al. (2019), the fault dip angle is expected to drastically decrease just below the brittle‐ductile transition. Below the detachments, which can be interpreted as ductile shear zones (Rabillard et al., 2018), an elasto‐plastic rheology can be assumed and rocks behave like viscoelastic materials (Carcione et al., 2014; Fayon et al., 2000). The brittle‐ductile transition in this sector of the Apennines deepens going from west to east (e.g., Carannante et al., 2013; Carminati et al., 2001): taking *η*_lc_ = 5 × 10^18^ Pa s and about 18–22 km of thickness for the lower crust, we obtain a displacement field consistent with the geodetic observations. Such setting (Figure 11b) is in line with model LC18 of Riva et al. (2007). We can further simplify such a model and take a constant brittle‐ductile transition at 15 km depth (Figure 11a) matching the near‐ and far‐field post‐seismic displacements slightly better. As shown in Riva et al. (2007), lowering *η*_lc_ below 10^18^ Pa s leads to a worsening of the fit, in agreement with our findings (Text S7). On the other hand, Riva et al. (2007) suggest a slightly thinner viscoelastic layer (about 12 km) that is likely compensated by their lower viscosity (*η* = 10^18^ Pa s). As a matter of fact, a trade‐off between the thickness of the viscoelastic layer and its viscosity exists. Pousse‐Beltran et al. (2020) modeled a viscoelastic relaxation following the Norcia earthquake as well, but they found an opposite vertical polarity (i.e., uplift) and therefore disregarded viscoelastic process as a possible driving mechanism for post‐seismic deformation. We impute this fact to the rheological profiles they implemented, that accounts for a ∂η/∂z<0 and, according to Hetland and Zhang (2014), if a high viscosity layer is placed between the elastic layer and the underlying substrates, the vertical post‐seismic and coseismic deformations exhibit an opposite polarity. Both our viscoelastic models consider the thick layer of seismicity that bounds the high‐angle normal faults to be elastic on the time scale of the analysis. Hetland and Zhang (2014) showed that if the co‐seismic rupture does not entirely break the seismogenic layer then the unruptured portion behaves like a viscoelastic material with very high viscosity. Our tests suggest that the lower bound for the viscosity of the volume of seismicity described by Vuan et al. (2017) is 10^20^ Pa s, and lower viscosities would produce unobserved effects at near‐field GPS stations.

In light of the considerations discussed in this section, we propose that viscoelastic relaxation of the lower crust may be a mechanism contributing to the measured post‐seismic displacements, but a deeper understanding is limited by several factors. For instance the length of the post‐seismic time series analyzed is short (about 2 years) with respect to the typical time scales of viscoelastic processes (∼5 years for the inferred viscosity of this area), bearing also in mind that moderate events (such as the Mw 6.5 Norcia earthquake) lead to deformation rates in the order of few mm/year (Riva et al., 2007). Furthermore, a major problem in understanding the post‐seismic response of the lithosphere to an earthquake is the non‐uniqueness of the explanation. Since the vbICA separates only one post‐seismic component, a clear distinction between afterslip and viscoelastic relaxation contribution proved to be a challenging task. We suggest that both afterslip and viscoelastic relaxation acted during the post‐seismic phase of the sequence and one should be careful in preferring one mechanism to the other because of the good agreement with the observations (Freed et al., 2006). The good reproduction of GPS displacements by afterslip is not surprising, as the inversion is not constrained by physical processes such as coseismic stress changes, whereas the misfit produced by the viscoelastic model can be explained by both the fact that it is not the only deformation mechanism active during the post‐seismic phase and by the simplifications made in our forward models with respect to the real Earth's case.

## 6 Conclusions

Exploiting GPS ground displacement time‐series we study the post‐seismic phase of the complex 2016–2017 Central Italy seismic sequence. We separate the post‐seismic tectonic deformation signal from other hydrological deformation signals, the first being mapped in one single component despite the occurrence of at least three mainshocks. We find evidence of displacement related to post‐seismic relaxation as far as ∼90 km from the epicentral area, up to offshore stations in the Adriatic Sea. We exploit the high accuracy and temporal resolution offered by continuous GPS measurements in order to investigate the different mechanisms that caused post‐seismic ground deformation. Afterslip, which produces surface deformations up to ∼4–5 cm, is by far the most important process, explaining the majority of the measured displacements cumulated in 28 months after the Amatrice mainshock. We find that taking into account only the faults that hosted the mainshocks of the sequence leads to a poorer fit to the displacements observed at GPS sites in the Paganica area and at far‐field sites, which drives our choice to invert for slip also on the Paganica fault and on a sub‐horizontal shear zone highlighted by seismicity, respectively. The fact that the Paganica fault (unruptured in the co‐seismic phase of the sequence) accommodated cm‐scale slip in the post‐seismic phase, mainly aseismically, highlights how interaction among faults has to be taken into account while attempting a deterministic modelization of the earthquake cycle.

Given the afterslip concentration at the base of the seismogenic faults, the different temporal evolution of geodetic deformation and cumulative number of aftershocks and the discrepancies between measured and afterslip‐modeled displacements, a viscoelastic contribution to the observed post‐seismic ground displacements cannot be ruled out. In particular, we deem the relaxation of the lower crust to be a contributing mechanism in the 2 years following the Amatrice‐Visso‐Norcia seismic sequence. Bearing in mind the limits of the data and of our interpretation, we propose an afterslip model that is consistent with the co‐seismic ruptures on the structures involved in the 2016–2017 seismic sequence. Furthermore, we provide some preliminary values of the viscosity and thickness of the lower crust, leaving further investigations to future studies, which might consider an afterslip + viscoelastic joint inversion of possibly longer time series.

## Supporting information

Supporting Information S1

## Data Availability

We use publicly available raw GPS data. Raw GPS displacement time series are available on https://doi.org/10.5281/zenodo.4633475; the GPS displacement time series used in input to vbICA are available on https://doi.org/10.5281/zenodo.4633412. The seismic catalog is taken from Michele et al. (2020) and available at https://doi.org/10.5281/zenodo.3712731. The precipitation, temperature, and river flow data used to implement the hydrological model are available at https://annali.regione.umbria.it/, http://www.sir.toscana.it/consistenza-rete, https://console.regione.marche.it/ (http://app.protezionecivile.marche.it/sol/indexjs.sol?lang=it), for the Umbria, Toscana, and Marche region, respectively. Data for the Abruzzo region are available under request to the REGIONE ABRUZZO “Dipartimento delle Opere Pubbliche, Governo del Territorio e Politiche Ambientali Servizio Programmazione Attività di Protezione Civile Ufficio Idrografico e Mareografico” (idrografico@regione.abruzzo.it). Extraterrestrial irradiance data are available at http://www.soda-pro.com/web-services/radiation/extraterrestrial-irradiance-and-toa. Drainage direction maps used to define river basins are available at www.hydrosheds.org/page/availability. Global data sets used for the hydrological load model are taken from https://grace.jpl.nasa.gov/data/get-data/jpl_global_mascons/ (GRACE), https://disc.gsfc.nasa.gov/datasets/GLDAS_NOAH025_3H_2.1/summary (GLDAS); http://loading.u-strasbg.fr (ERA‐interim model); mean monthly temperature: GHCN Gridded data provided by the NOAA/OAR/ESRL PSL, Boulder, Colorado, USA, from their Web site at https://psl.noaa.gov/. A version of the vbICA software modified to take into account missing data is available at the Zenodo repository https://doi.org/10.5281/zenodo.4322548; the MATLAB code used to compute the TWS can be found at http://dx.doi.org/10.17632/m5p5xmrr7k.1. The software Relax used to model the viscoelastic relaxation is available at https://geodynamics.org/cig/software/relax/.
