# Supplementary material for: Post‐Seismic Deformation Related to the 2016 Central Italy Seismic Sequence From GPS Displacement Time‐Series
Source: J Geophys Res Solid Earth. 2021 Aug 29;126(9):e2021JB022200. doi: 10.1029/2021JB022200 (PMC9285078; doi:10.1029/2021JB022200)
Supplement: Supplementary file 1 — Supporting Information S1 [file JGRB-126-0-s001.pdf]

Journal of Geophysical Research: Solid Earth

Supporting information for

Post-Seismic Deformation Related to the 2016 Central Italy Seismic Sequence from GPS Displacement Time-Series

E. Mandler[1], F. Pintori[2], A. Gualandi[2], L. Anderlini[3], E. Serpelloni [2,3] and M. E. Belardinelli[1]

1: Dipartimento di Fisica e Astronomia "Augusto Righi", Alma Mater Studiorum Università di Bologna, Italy

2: Istituto Nazionaledi Geofisica e Vulcanologia (INGV), Osservatorio Nazionale Terremoti, Italy

3: Istituto Nazionale di Geofisica e Vulcanologia (INGV), Sezione di Bologna, Italy

Contents of this file

Text S1 to S10

Figures S1 to S20

Tables S1 to S3

Introduction

The supporting information details some methodology and presents some intermediate results.

S1. GNSS data analysis

S.1.1. GNSS dataset and data processing

The position time-series have been obtained adopting a three-step procedure approach, as in Serpelloni et al. (2006, 2018), that includes: 1) raw phase data reduction, 2) combination of loosely constrained network solutions and reference frame definition and 3) time-series analysis, including velocity estimates and spatial filtering of common mode errors.

The raw GPS observables have been analyzed using the 10.70 version of the GAMIT/GLOBK package (Herring et al., 2018) adopting standards defined in the framework of the IGS “Repro2 campaign” (http://acc.igs.org/reprocess2.html). The GAMIT software is used to estimate station positions, atmospheric delays, satellite orbits, and Earth orientation parameters from ionosphere-free linear combination of GPS phase observables using double differencing techniques to eliminate phase biases caused by drifts in the satellite and receiver clock oscillators. GPS pseudo-range observables are used to constrain clock timing offsets and to improve automated editing of the phase data, assisting in the resolution of integer phase ambiguities. GPS phase data are weighted according to an elevation-angle-dependent error model (Herring et al., 2018) using an iterative analysis procedure whereby the elevation dependence is determined from the observed scatter of phase residuals. In this analysis the satellites orbit parameters are tightly constrained to the IGS final products. We use the IGS absolute antenna phase center model for both satellite and ground-based antennas, which improves the accuracy of estimates for the vertical components of site position by mitigating reference frame scale and atmospheric mapping function errors (Schmid et al., 2005, 2007). While the first-order ionospheric delay is eliminated by the ionosphere-free linear combination, the second-order ionospheric corrections are applied based on the formulation of (Petrie et al., 2010), using IONEX files from the Center for Orbit Determination in Europe (CODE). The tropospheric delay is modeled as piecewise linear model and estimated using the Vienna Mapping Function 1 (VMF1; Boehm et al., 2007) with a 10° cutoff. We use the Global Pressure and Temperature 2 (GPT2; Lagler et al., 2013) model to provide a priori hydrostatic delays. The pole tide was also corrected in GAMIT by IERS standards. The Earth Orientation Parameters (EOP) are tightly constrained to priori values obtained from IERS Bulletin B. Non-tidal atmospheric loading and ocean tidal loading

are corrected using MIT filtered atmospheric displacements files (available at ftp://everest.mit.edu/pub/GRIDS) and the FES2004 (Lyard et al., 2006) model, respectively. The International Earth Rotation Service (IERS) 2003 model for diurnal and semidiurnal solid Earth tides was set. Because of the large number of stations included in our Euro-Mediterranean GPS processing (~3000), this step is performed for several sub-networks, each made by <50 stations, with each sub-network sharing a set of high-quality IGS stations, which are used as tie-stations in the combination step.

In the second step we use the ST_FILTER program of the QOCA software (http://qoca.jpl.nasa.gov), which adopts a Kalman filter estimation algorithm (Dong et al., 1998, 2002), to combine all the daily loosely constrained solutions with the global solution of the IGS network made available by MIT (http://sopac.ucsd.edu), and simultaneously realize a global reference frame by applying generalized constraints (Dong et al., 1998). Specifically, we define the reference frame by minimizing the velocities of the IGS core stations (http://igscb.jpl.nasa.gov), while estimating a seven- parameter transformation with respect to the GPS realization of the ITRF2008 frame (Altamimi et al., 2011), i.e., the IGb08 reference frame.

In the third step we analyze the position time series in order to perform de-trending and filtering of common mode noise signals, and realize the displacement time-series to be used as input of the vbICA (see Section 2.2). Because of the presence of non-linear signals (i.e., the post-seismic transients) and of short time-series (e.g., those installed soon after the Amatrice mainshock) for which the linear tectonic trend is difficult to determine, we use a constrained non-linear least-squares estimator, with linear trends constrained to apriori values. We model the time-series with a classic trajectory model (Bevis & Brown, 2014) estimating for offsets due to stations equipment changes and earthquakes, annual and semi-annual periodic signals, a linear velocity term and an exponential term describing the post-seismic transient displacements. Remarkably, in order to remove the linear trend from short time series (with the first epoch after 2013), an interpolated velocity field was constructed by modeling the velocities of those stations having time series longer than 5 yrs adopting a multiscale approach (Tape et al., 2009). These modeled values are used as a-priori velocities in the time-series analysis. Hence, for short time series, the linear trend was constrained to be close to the a-priori value (in a range of ±30% for the horizontal components, and ±50% for the vertical component); whereas for long time series (first epoch prior to 2013) it is left as a free parameter. In order to better assess the offsets, the estimate is forced to be close to the difference between the median value of the few positions after the jump and the median value of the few positions prior to it in a range of ± the mean value

of displacement errors. This can help in better constraining the offset that could otherwise affect the post-seismic assessment. The model derived from the combination of these signals (red lines in Fig. S1) is then subtracted from the position time series in order to get the residual positions. The residual time-series are then used to estimate the Common Mode Error (CME) performing a Principal Component Analysis (PCA), as described in Dong et al. (2006). The PCA is performed at a continental-scale, over the same area used by Serpelloni et al. (2013), and the first two PCs are here considered as CME. Moreover, during this step, all GPS stations interested by past earthquakes have been excluded from the PCA. This prevents the removal of the eventual more localized signals of geophysical interests recorded by the GPS stations in the study region, since the PCA detects the signals common to a much larger region. As a result, after removing the CME, the typical repeatability in our analysis is ~1 mm for the horizontal components, and ~3 mm for the vertical component, with a 30% gain in the daily repeatability and a significant improvement of the signal to noise ratio. After the spatial filtering, the estimated seasonal motions are added back to the filtered time-series, obtaining position time series with a reduced scatter around the adopted model which are used as input of the blind source separation analysis performed with the vbICA method (see Section 2.2).

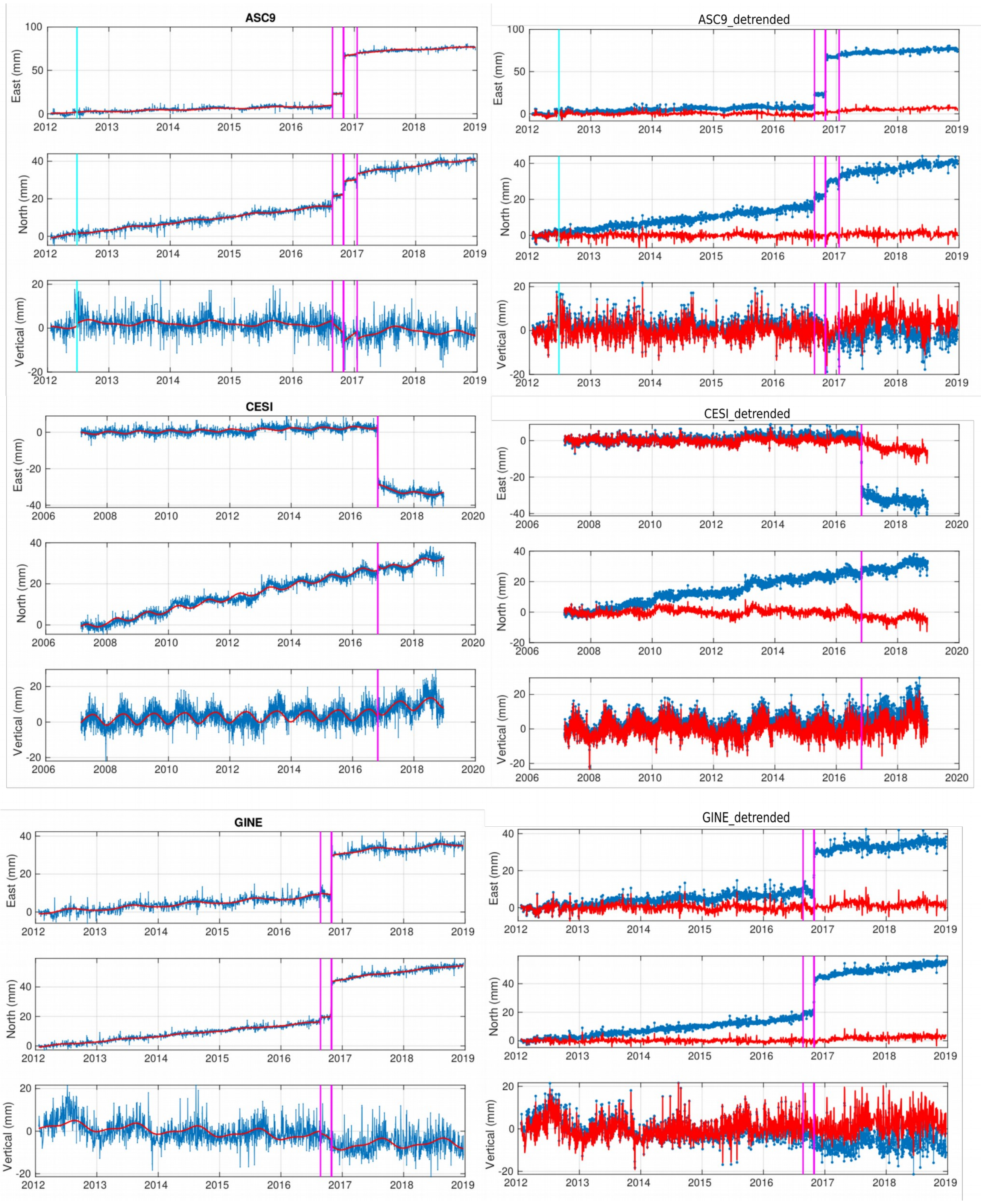

Figure S1. Left column panels show an example of fit to the time-series of ASC9, CESI and GINE sites (red lines); right column shows the raw time-series (light blue curves) and the detrended time-series (red curves) for the same sites. Magenta vertical lines mark the earthquakes epoch, whereas light blue vertical lines the instrumental offsets epoch.

S.1.2. ICA uncertainty

In this section we describe the novel procedure adopted in this study to associate a more realistic uncertainty with the independent components. We proceed as follows: we perform 100 ICA decompositions, each time randomly perturbing the original GPS time series assuming a nominal Gaussian uncertainty at each available epoch. We refer to these 100 decompositions as ICA_{rand}. Differently from the more common Principal Component Analysis (PCA), a problem with the ICA is that the ordering of the ICs is not well defined. Fortunately, the extracted ICs are sufficiently robust with respect to the random perturbations imposed, and we can thus sort the ICs ordering them on the base of the correlation between their temporal sources and the original sources obtained not perturbing the data (i.e. the decomposition shown in Figures 3A and 4 of main text). We estimate the uncertainty on the spatial pattern U, the weights Sigma and the temporal functions V considering how spread their values are across the 100 decompositions. In practice, we calculate the sample variance for each element of each matrix. This procedure provides larger uncertainties with respect to those outputted by the vbICA code, and we consider them to reflect more realistically the uncertainty in the data.

S2. Pre-seismic analysis

In this section we show the components retrieved by the analysis of the pre-seismic phase of the Amatrice-Visso-Norcia seismic sequence. The time dependence of the independent components (Fig. S2 panel a, b, c, d) does not highlight the occurrence of any geodetic transient; their spatial response (lower panel) is generally sparse and it does not support the presence of any ongoing localized tectonic process in the preparatory phase of the 2016-2017 seismic sequence.

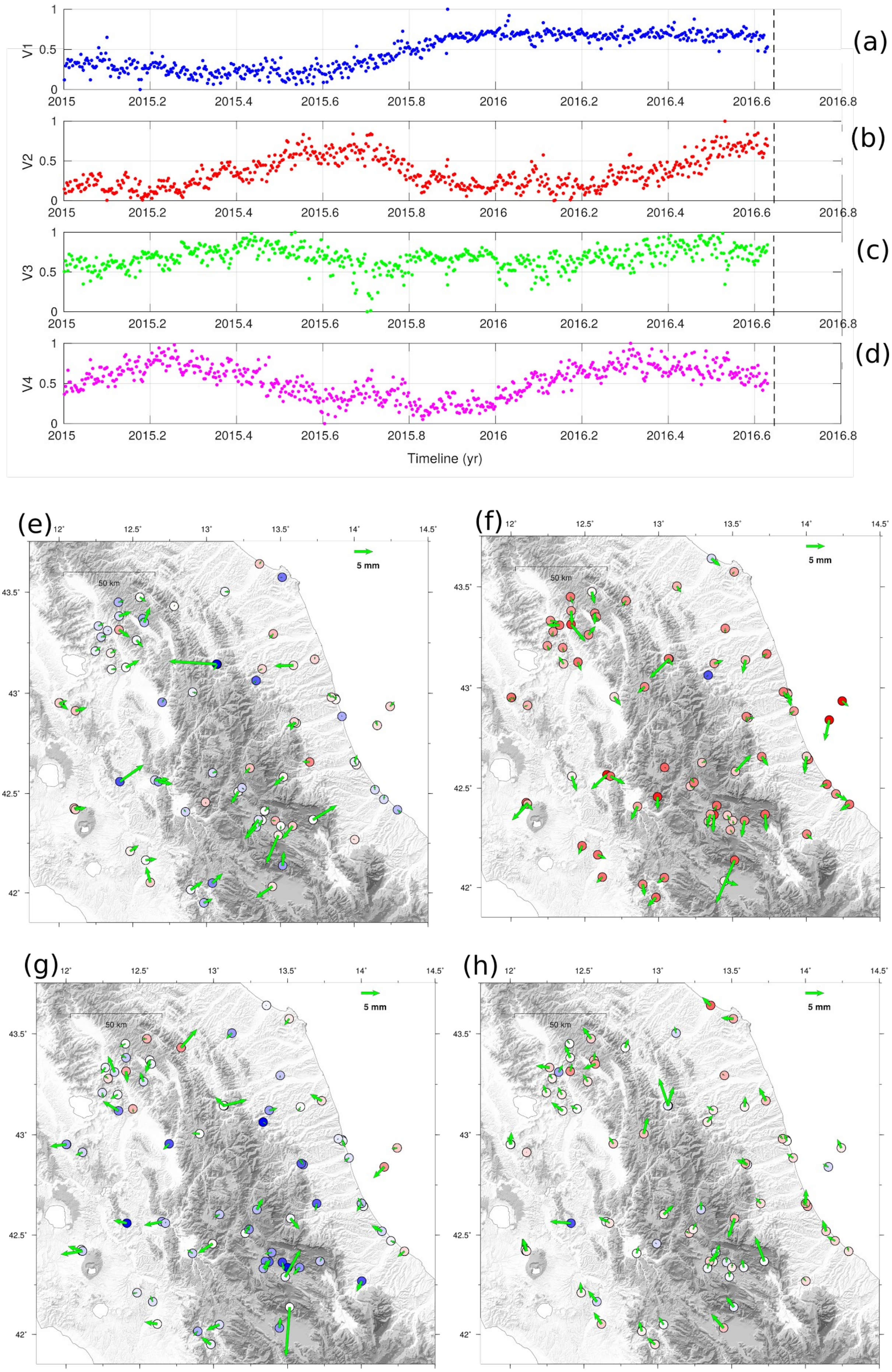
(a)
(b)
(c)
(d)
V1
V2
V3
V4
0
0.5
1
2015
2015.2
2015.4
2015.6
2015.8
2016
2016.2
2016.4
2016.6
2016.8
Timeline (yr)
(e)
(f)
(g)
(h)
12˚
12.5˚
13˚
13.5˚
14˚
14.5˚
42˚
42.5˚
43˚
43.5˚
50 km
5 mm

Fig. S2. Temporal evolution and dimensional spatial response of IC1 (a, e), IC2 (b, f), IC3 (c, g), IC4 (d, h) of the analysis on the pre-seismic phase (time span 2015-2016.64). Vertical dashed lines in panels (a, b, c, d) mark the 24th of August mainshock. In the lower panels the spatial responses to the sources of deformation are given in mm.

S3. Hydrological analysis

Figure S3 shows the 5 hydrological basins considered, defined using the drainage direction maps (www.hydrosheds.org/page/availability) and watershed outlets located at the river discharge measurements on the Tevere, Nera, Tronto, Pescara and Aterno rivers.

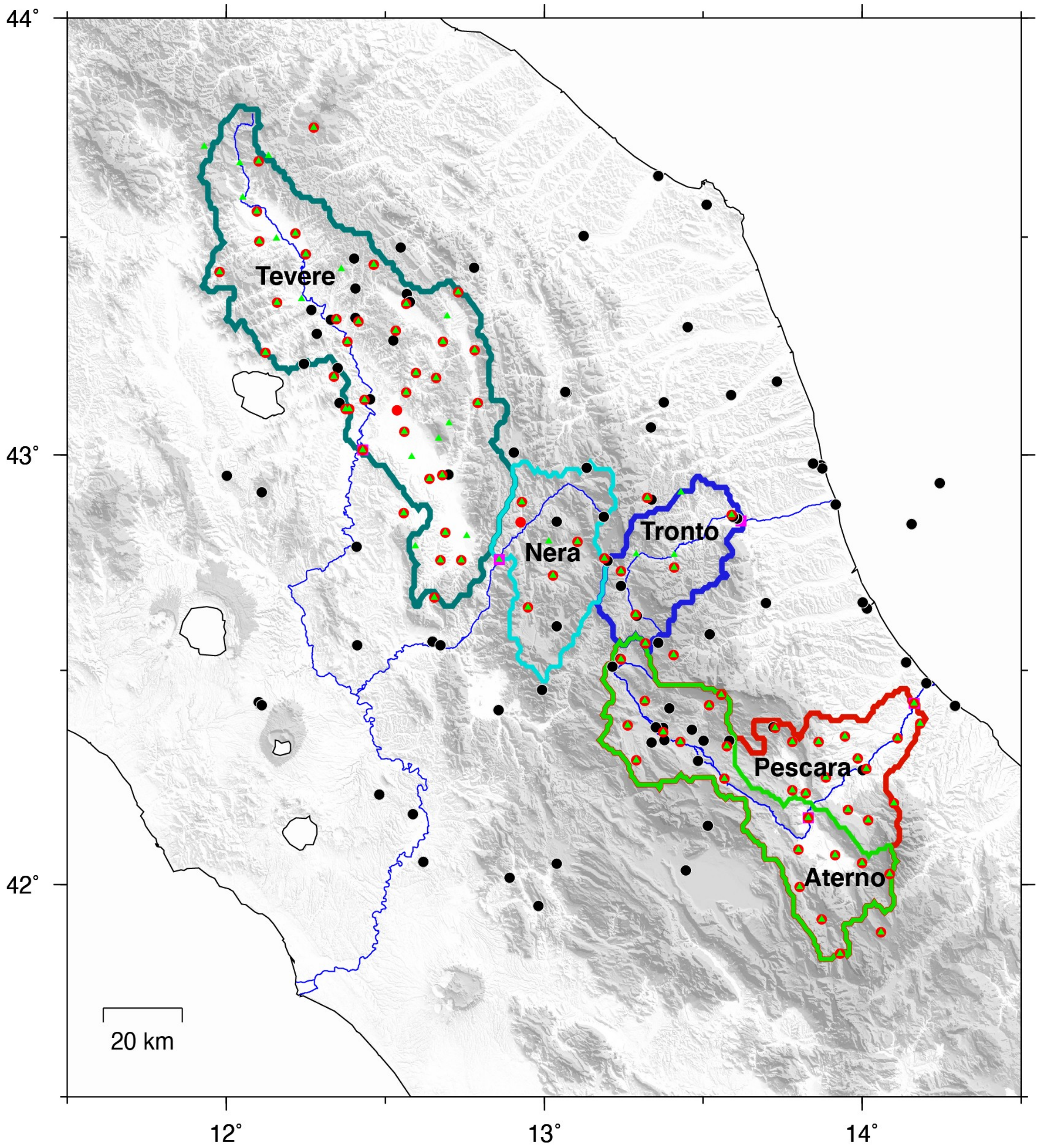

Figure S3. Hydrological basins of the Tevere (dark green), Nera (cyan), Tronto (blue), Aterno (light green), Pescara (red) rivers. Black dots: GNSS stations; purple squares: river gauging stations; green triangles: pluviometers; red circles: thermometers.

The Tables S1 and S2 show the cross-correlation between TWS and V3, V4 (Fig. 6 main text) respectively. The time lag that maximizes the correlation is reported, too.

Hydrological Basin	Pearson Correlation Coefficient TWS - V3	Lag (days)
Tevere	0.7077	110
Tronto	0.7528	93

Pescara	0.7374	79
Aterno	0.7606	63
Nera	0.7404	82

Table S1. Pearson cross-correlation coefficient between TWS computed in the hydrological basins and V3 (Fig. 6 main text). TWS anticipates V3 by a number of days estimated in the third column. Both TWS and V3 have been detrended.

Hydrological Basin	Pearson Correlation Coefficient TWS - V4	Lag (days)
Tevere	0.4831	20
Tronto	0.4144	8
Pescara	0.3043	6
Aterno	0.2128	9
Nera	0.2163	10

Table S2. Pearson cross-correlation coefficient between TWS computed in the hydrological basins and V4 (Fig. 6 main text). TWS anticipates V4 by a number of days estimated in the third column. Both TWS and V4 have been detrended.

S4. Post-seismic re-analysis

In this section we show the results for the vbICA performed on the residual time series where the hydrological components, described in Section 3 of the main text, are removed from the original time series. Since we are focusing our attention on the post-seismic phase only, we analyze the time span 2016-2019, and the vbICA is performed by fixing the number of ICs L=3 as suggested by an F-test. In the following images we show the results of this decomposition: the post-seismic relaxation is still clear (IC1, Fig. S4) and it explains the majority of the variance of the data (S_1=1523 mm); the second component (IC2, Fig. S5) shows a non monotonic evolution that does not match with what we observe in the post-seismic time series. Moreover its relative importance in explaining the data variance is limited (S_2=403 mm), therefore we neglect it as a contributing source of the post-seismic relaxation. The IC3 (Fig. S6) shows a periodical behaviour in its temporal part and for this reason we consider it as due to incomplete correction of the hydrological signals.

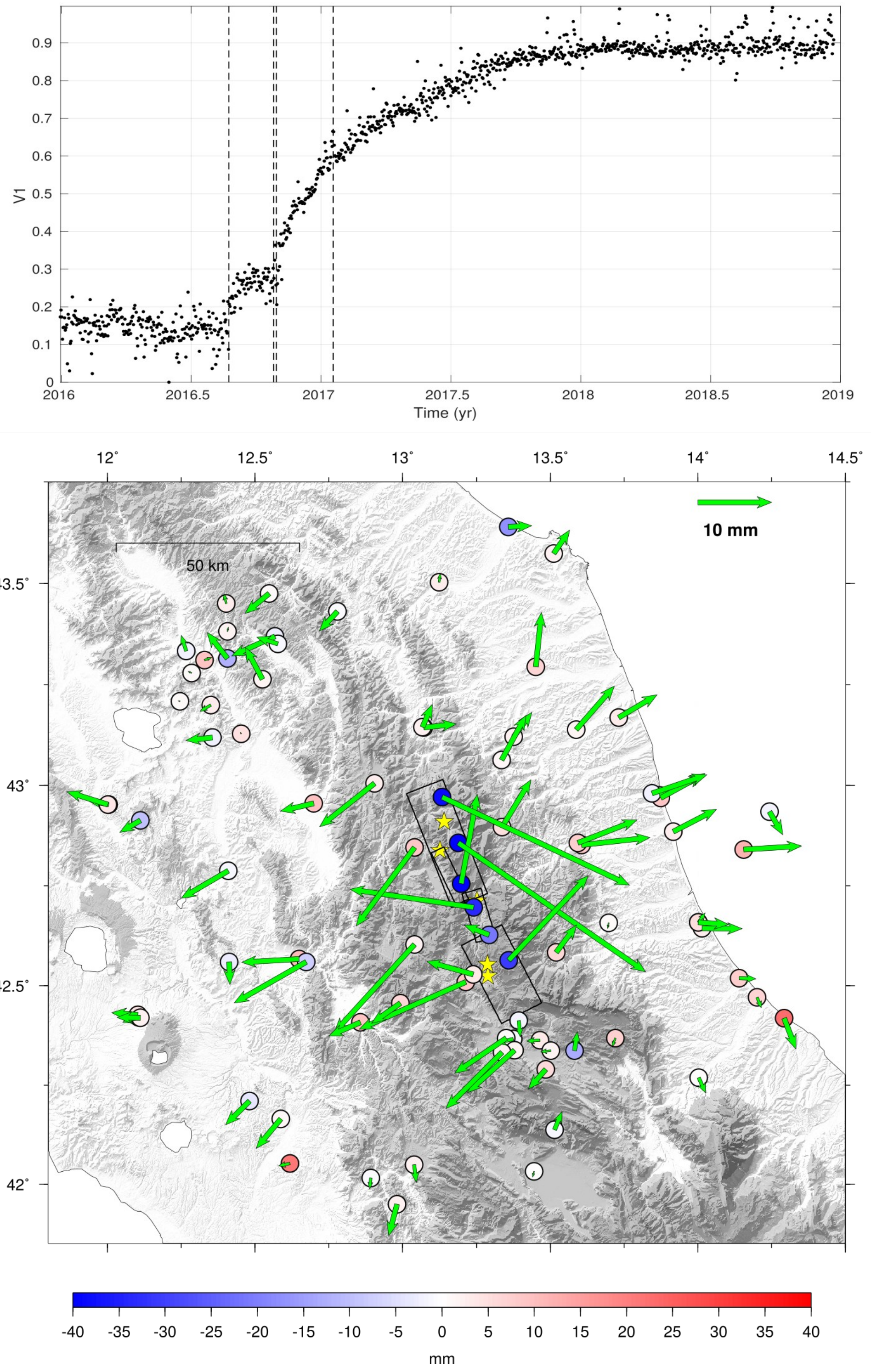

Fig S4. The IC1 (upper panel = temporal evolution, lower panel = spatial pattern) of the analysis on the post-seismic phase of the time series filtered from the hydrological components. Yellow stars show the epicenters of the mainshocks while the black boxes show the location of the faults responsible for the 2016-2017 sequence as in Cheloni et al. (2017, 2019).

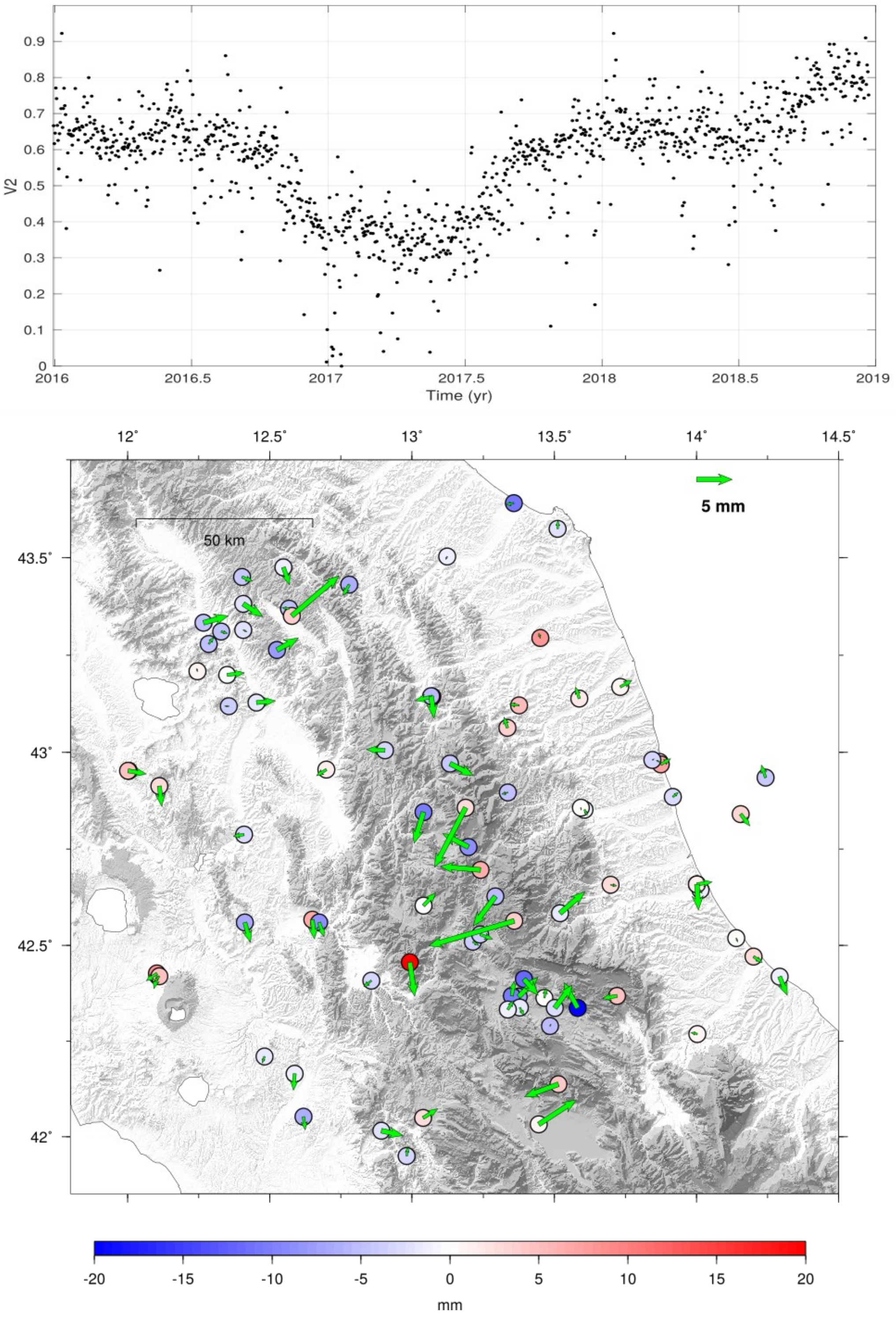

Fig S5. The IC2 (upper panel = temporal evolution, lower panel = spatial pattern) of the analysis on the post-seismic phase of the time series filtered from the hydrological components.

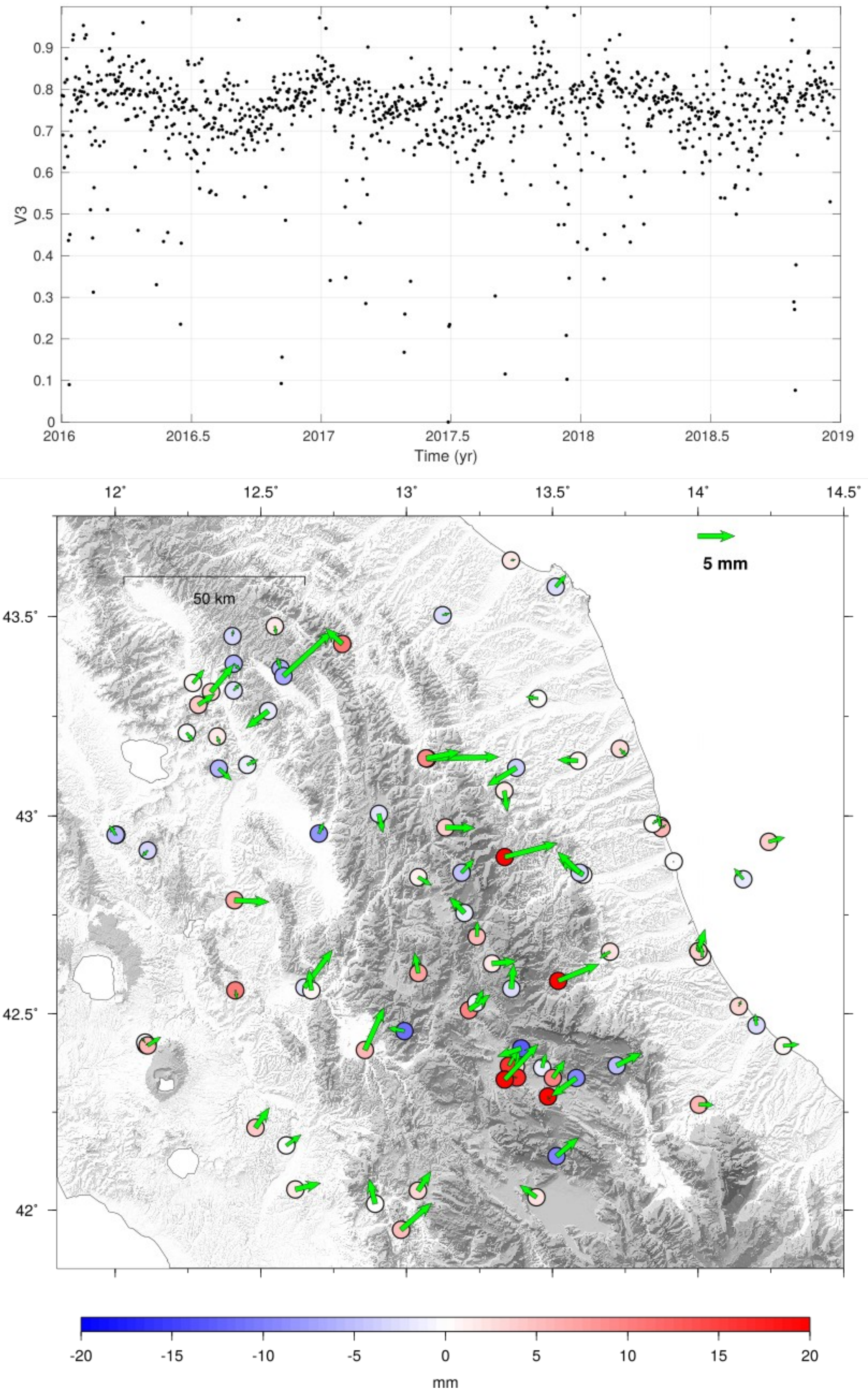

Fig S6. The IC3 (upper panel = temporal evolution, lower panel = spatial pattern) of the analysis on the post-seismic phase of the time series filtered from the hydrological components.

S5- Paganica sites: post-seismic separation and effects on the slip inversion

In this section we show the importance of a correct separation among tectonic and non-tectonic sources for the sites in the Paganica area, and how the post-seismic displacement associated with these sites implies the inclusion of the Paganica fault in the inversion. GPS stations in the Paganica area are heavily affected by the IC3 (Fig. 4 main text) as it is quite clear from the raw time-series (Fig. S7); however to neglect a post-seismic contribution to the total displacement leads to a bad data modelization (fig. S7). To double check this fact we subtracted the hydrological ICs from the raw data. The residuals show a mm-scale post-seismic transient (fig. S8) consistent with the spatial displacement associated with the IC1 (fig. 4a main text). Once we have validated the separation of the post-seismic displacement associated with the sites in the Paganica area, we show that such displacement requires to include the Paganica fault in the inversion, which is carried out following the procedure described in Section 4.1 of main text. If the Paganica fault is not included, we notice from Figure S9a the strong concentration of slip (up to 35 cm) on the southern edge of the Campotosto fault which is likely driven by the position of the GPS sites with respect to the fault. Despite the presence of such concentration of slip, we notice that the displacement at the Paganica sites is largely underestimated (Fig. S9b).

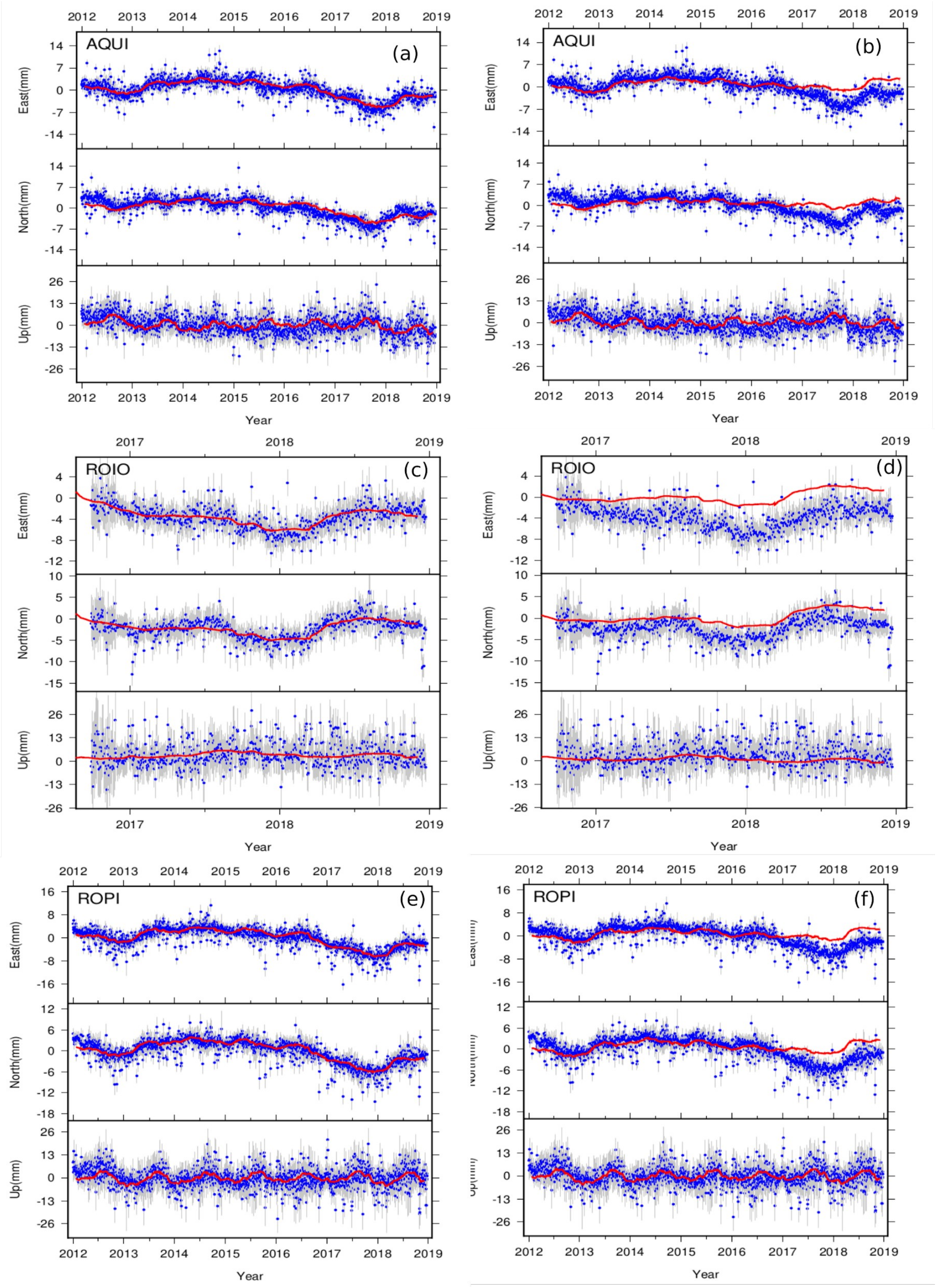

Fig S7. Comparison of AQUI, ROIO, ROPI time-series reconstruction using all of the ICs (panels a, c, e) and using only the non-tectonic components (panels b, d, f). Blue dots show the raw data while red lines the ICA modelization.

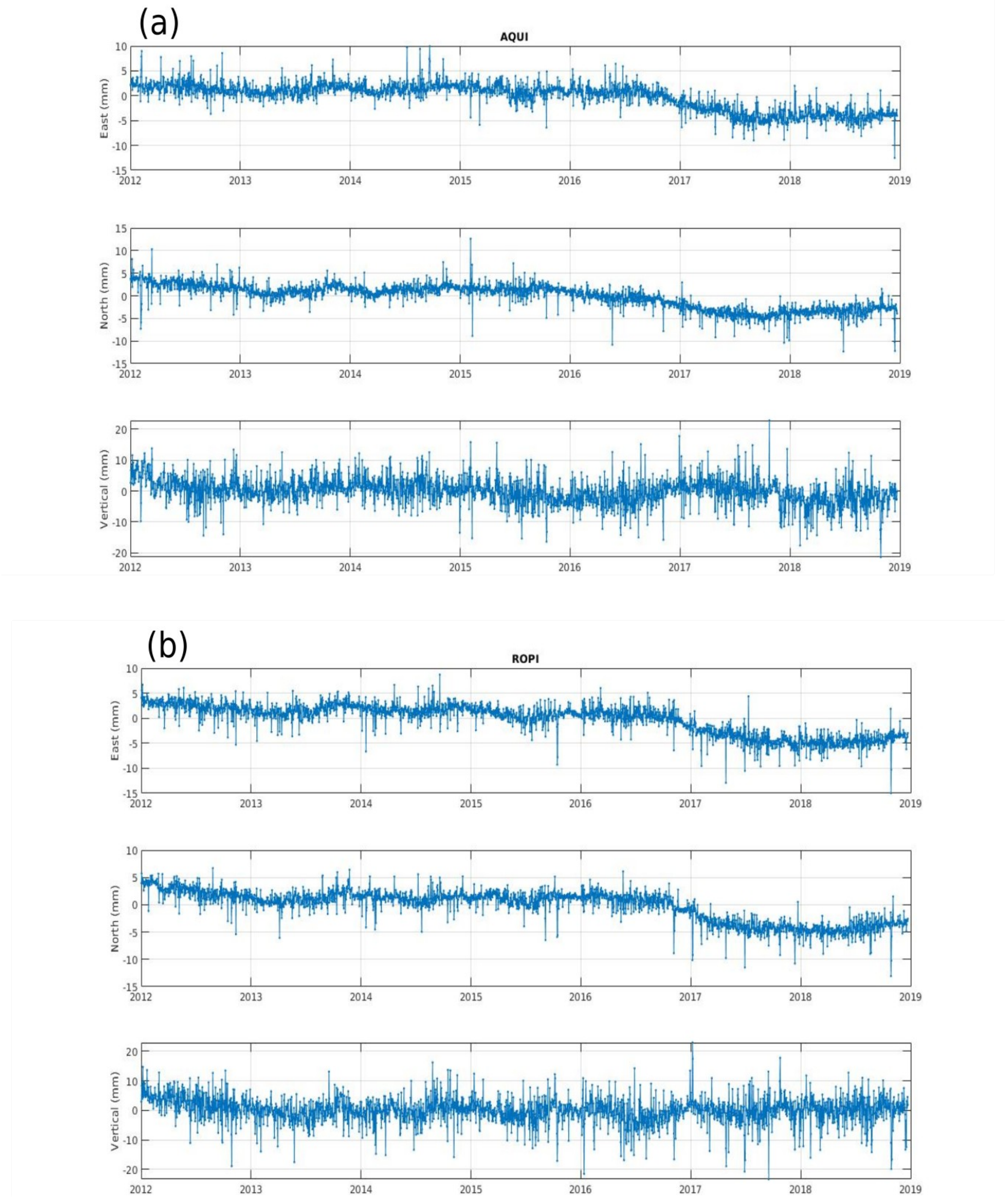

Fig S8. In figure the residuals among the raw time series and the IC 2, 3, 4, for the GPS stations AQUI (a), ROPI (b). In the post-seismic phase they show a mm-scale deformation prevalently SW-oriented, consistent with the direction and intensity of the spatial part of the IC1.

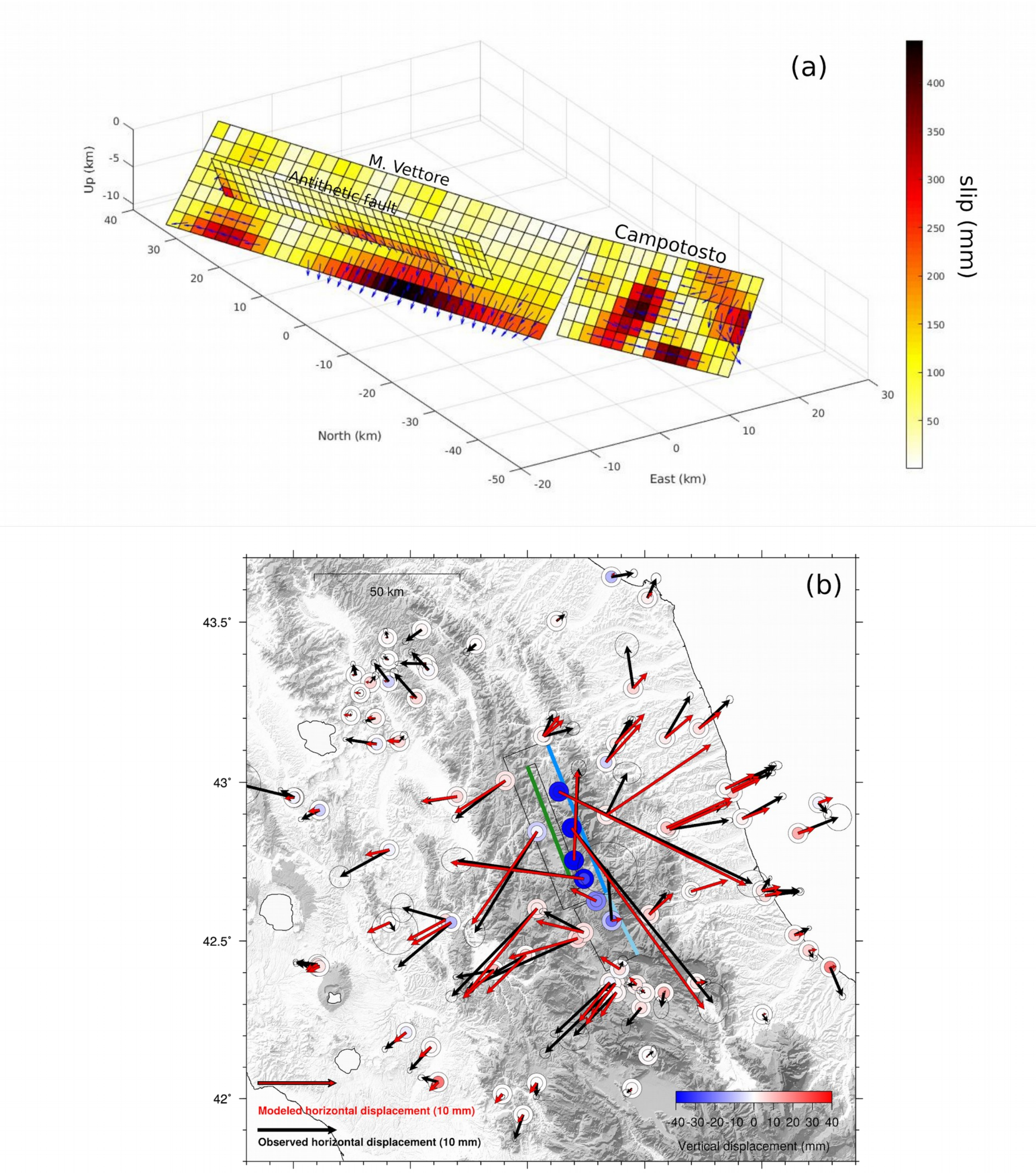

Figure S9. (a): Slip distribution on the M. Vettore fault, the antithetic fault and the Campotosto fault; (b): map of the data modelization for the inversion with the Paganica fault not included. Faults' traces are colored as in Figure 8 of the main text.

S6. Far- and Near-field separation

To discriminate between near and far fields, namely which stations are more affected by slip on

faults, we proceed as follows. We solve the forward problem relative to a 60 km long, 10 km deep, rectangular fault plane uniformly slipping by 1 m and embedded in a homogeneous elastic half-space. This dislocation represents an along-strike extension of the major structures described in Cheloni et al. (2017, 2019), centered on the seismicity pattern that followed the seismic sequence (Figure S10a). The calculated displacement at the GPS locations basically consists in the Green's function response, and it is made of a three components vector per station j: . $Gj=[G_{je}, G_{jn}, G_{ju}]^T$, j=1, ..., N_{stn} . We compare the L2 norm of such vector normalized by the maximum value retrieved for all the stations, $gj = |Gj| / max\{|Gi|\}_{i=1}^{Nstn}$ for j=1,..,N_{stn},, with the normalized L2 norm relative to the spatial post-seismic response at the studied stations, $uj = |Uj| / max\{|Ui|\}_{i=1}^{Nstn}$ for j=1,..,N_{stn}. In order to better identify the GPS sites that are most affected by the slip on the fault (i.e. near field stations) we consider a local reference frame with origin in the center of the rectangular plane used for the forward model. We define the horizontal plane by the x-axis parallel to the fault strike and the y-axis perpendicular to it. In Figure S10b we plot g (blue) and u (orange) with respect to the distance (from the origin) normalized to a characteristic length for x-axis (the half length of the fault trace, i.e. 30 km) and the y-axis (two times the depth of fault, i.e. 20 km). This normalization is chosen to take into consideration not only the main deformation signal along the extensional direction, but also a possible heterogeneous elastic response along the strike direction due to the complex faults system involved. We observe that the two signals show spatial decays that differ from each other for normalized distances greater than 2. For such distances u is systematically higher than the elastic response g, suggesting that the displacement recorded at these GPS sites cannot be described solely by afterslip. We therefore consider this threshold value of normalized distance equal to 2 in order to distinguish GPS sites into two groups: i) the near field group, for distances less than the threshold value and ii) the far field group, for greater distances (Figure S10).

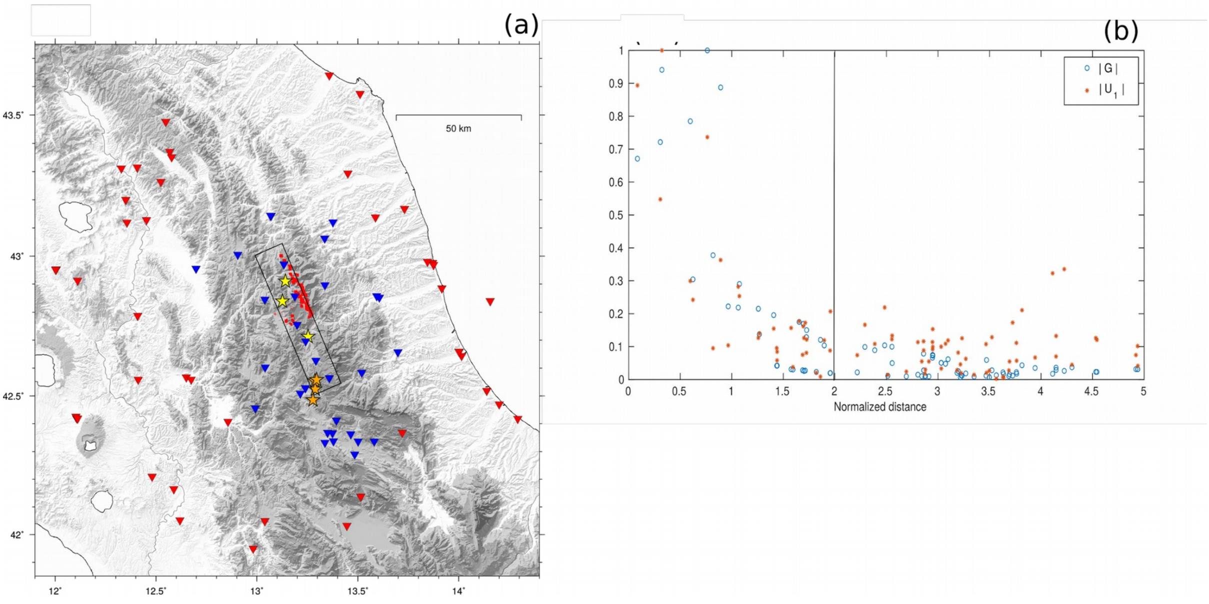

Figure S10. Panel (a) shows the near field and far field GPS stations' position (respectively blue and red triangles), the Amatrice, Visso and Norcia earthquakes (yellow stars) and the January 2017 Campotosto events (orange stars). The black rectangle represents the fault used to distinguish stations in the near field from those in the far field. Panel (b) shows g (blue circles) and u (orange dots) vs the normalized distance. The vertical line marks the threshold distance.

S7. Viscoelastic time series

In this section we show the time series obtained from the Relax simulation which considers a viscoelastic lower crust with $\eta_{lc}=10^{17}$Pa s. In particular we show in map the deformation pattern after 2 years from the 30th of October mainshock, and we report the results for some of the sites with a non-monotonic evolution in time.

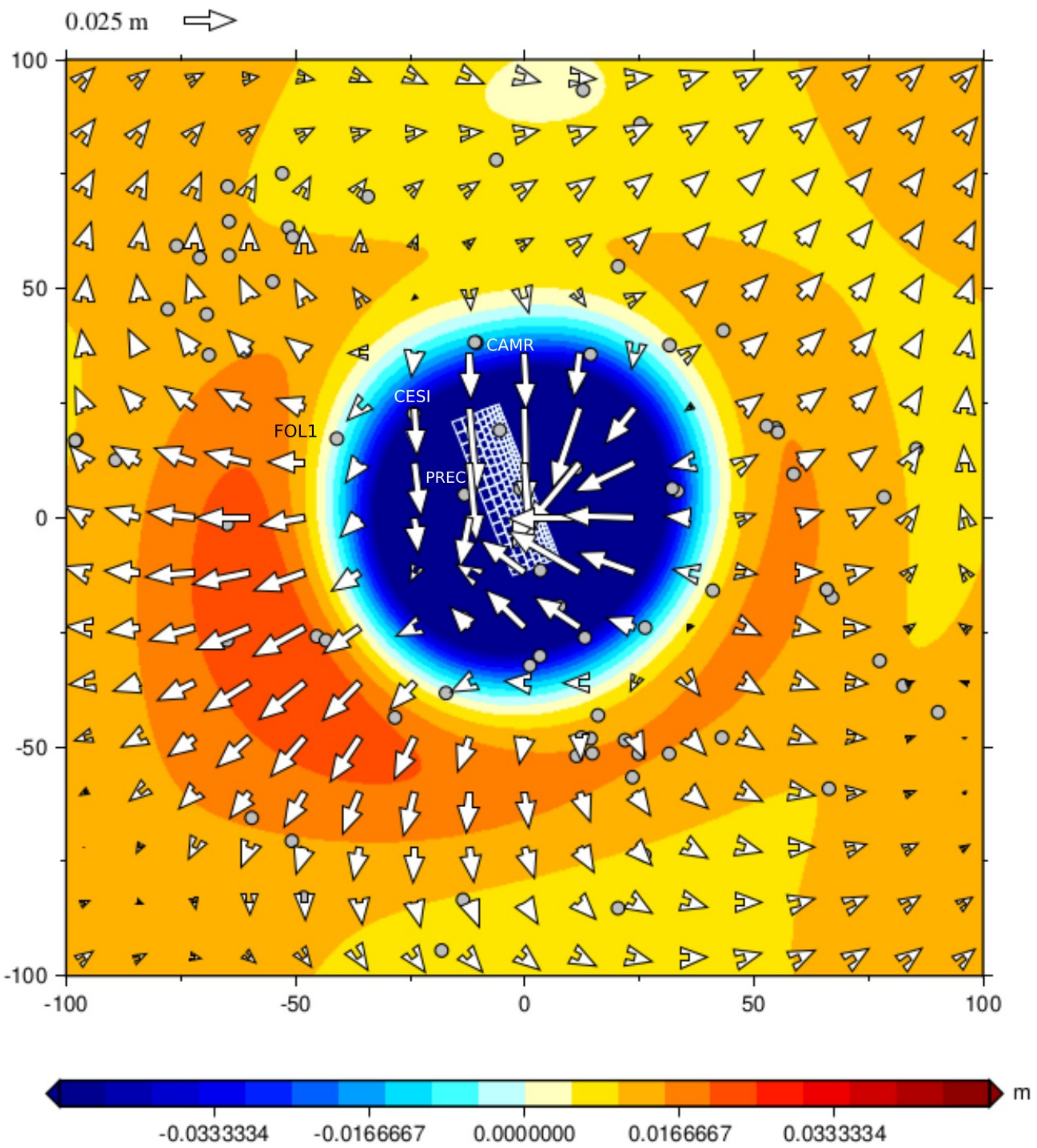

Fig S11. In map the viscoelastic displacement pattern deriving from the model with $\eta_{lc} = 10^{17}$ Pa s 2 years after the Norcia mainshock. White grid shows the source fault as in Cheloni et al. (2019), grey circles mark the position of the GPS sites. Lengths along the x and y-axis are in km from the origin (lon=13.2°, lat=42.8°).

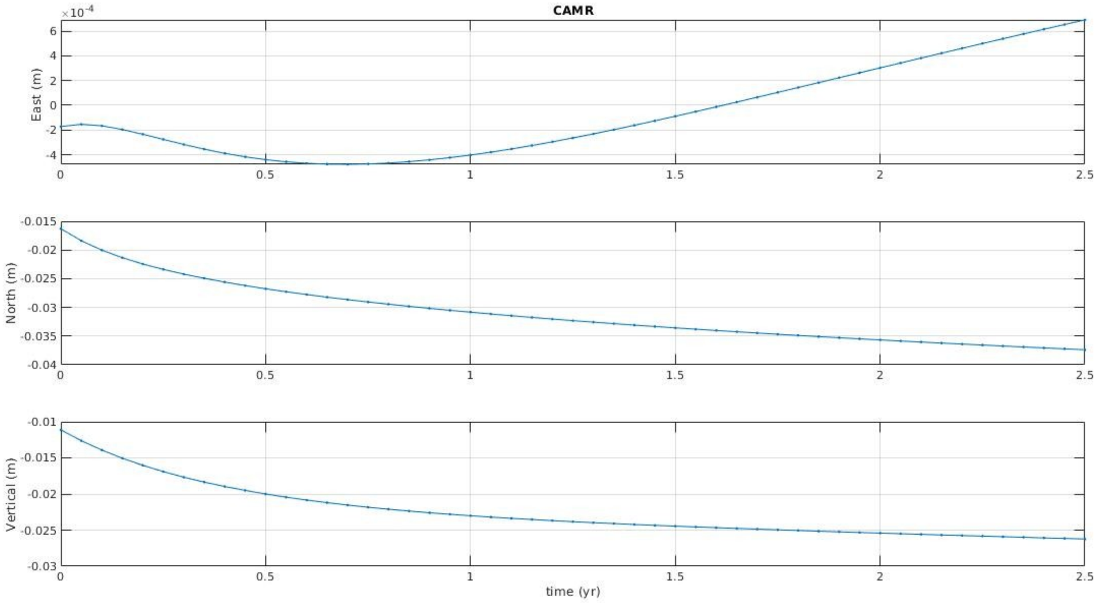

Fig S12. In figure the time series for CAMR GPS site. Displacement is in meters while time is in years measured from the Norcia earthquake epoch.

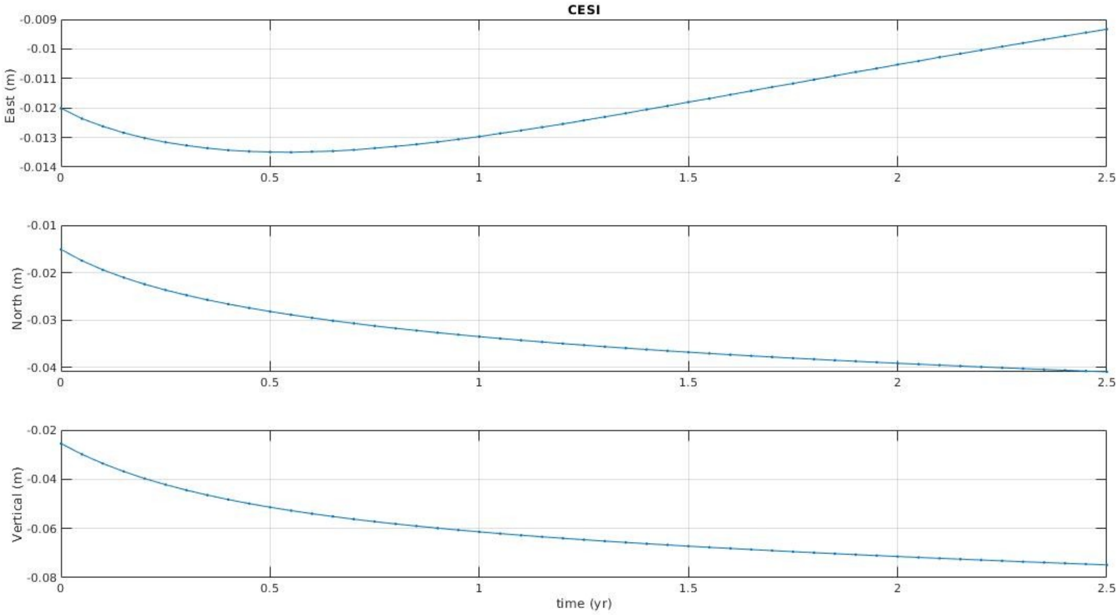

Fig S13. In figure the time series for CESI GPS site. Displacement is in meters while time is in years measured from the Norcia earthquake epoch.

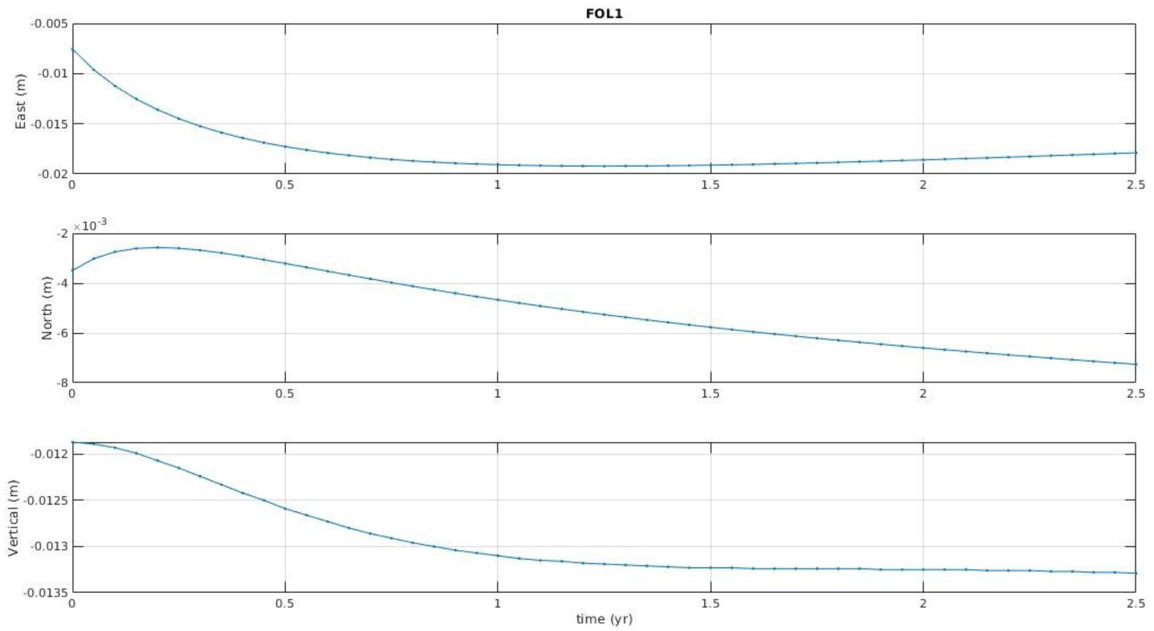

Fig S14. In figure the time series for FOL1 GPS site. Displacement is in meters while time is in years measured from the Norcia earthquake epoch.

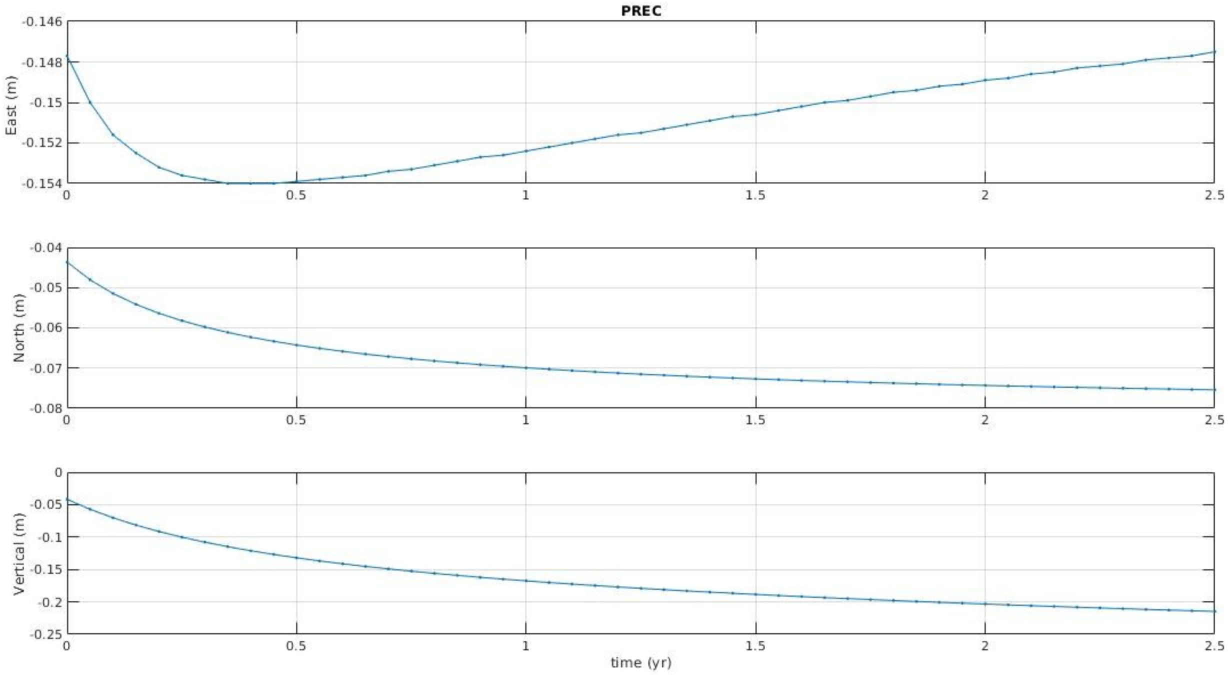

Fig S15. In figure the time series for PREC GPS sites. Displacement is in meters while time is in years measured from the Norcia earthquake epoch.

S8. Comparison between the afterslip models

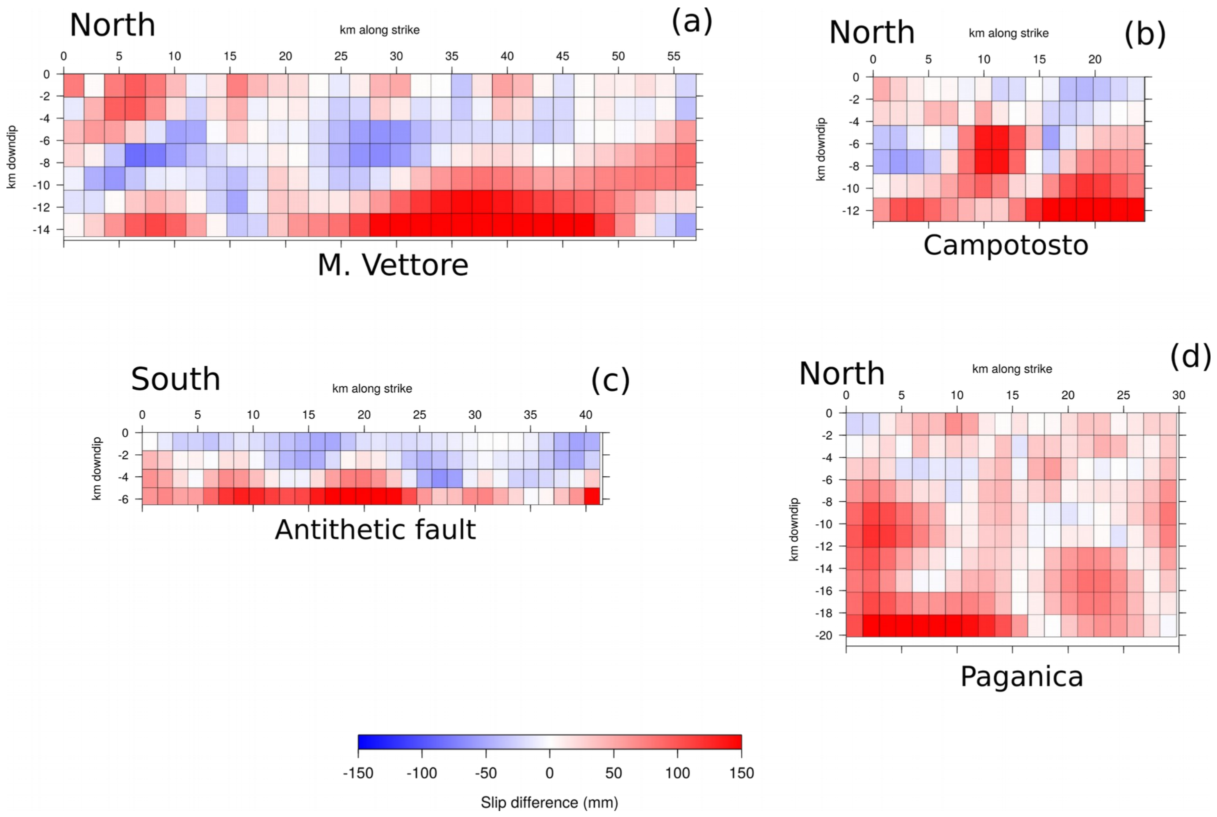

Fig S16. Figure shows the difference of slip magnitude (in mm) between the afterslip solutions of Section 4.2 and 4.3, main text, in a strike-dip reference system for (a) the M. Vettore, (b) the Campotosto, (c) the antithetic fault and (d) the Paganica fault.

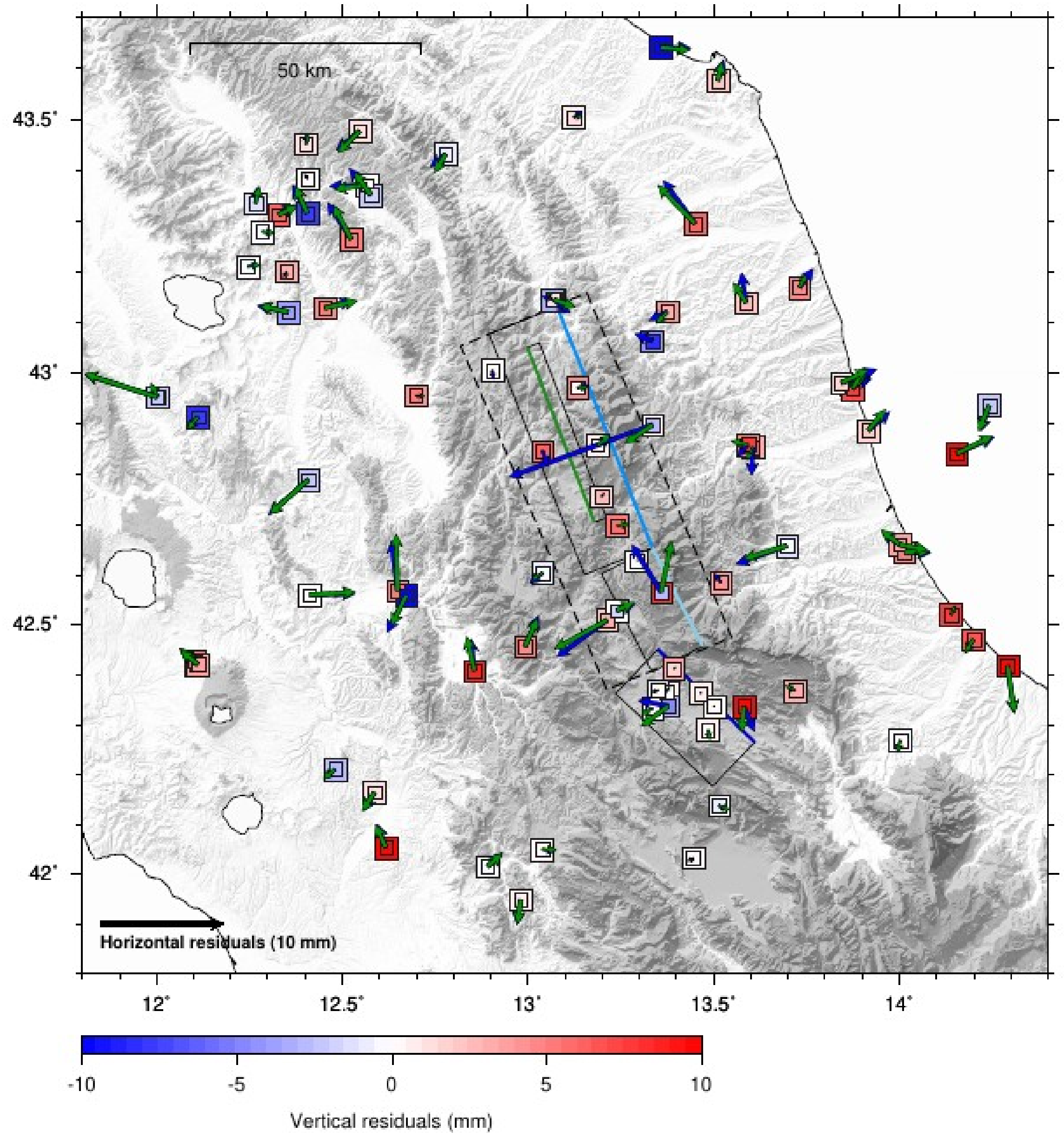

Fig S17- In map the residuals between the observed and the modeled horizontal (arrows) and vertical (squares) components of the post-seismic cumulative displacement are shown. Blue arrows and inner squares are for the model without the shear zone; green arrows and outer squares for the model with the shear zone included.

Model	$WRMSE^{TOT}$ (mm)	$WRMSE^{E}$ (mm)	$WRMSE^{W}$ (mm)	$WRMSE^{N.F.}$ (mm)
Section 4.2	429	181	181	67
Section 4.3	405	151	180	74

Tab S3. Weighted root mean square error (WRMSE) for the two afterslip models described

in Section 4.2 and 4.3, main text. WRMSE are computed on the cumulative post-seismic displacement in the time span 25th of August 2016 - 2019 on the whole dataset ($WRMSE^{TOT}$), on the two subsets of GPS stations east and west of the fault system (respectively $WRMSE^{E}$ and $WRMSE^{W}$) and on the near field GPS stations ($WRMSE^{N.F.}$).

S9. Inversion Resolution

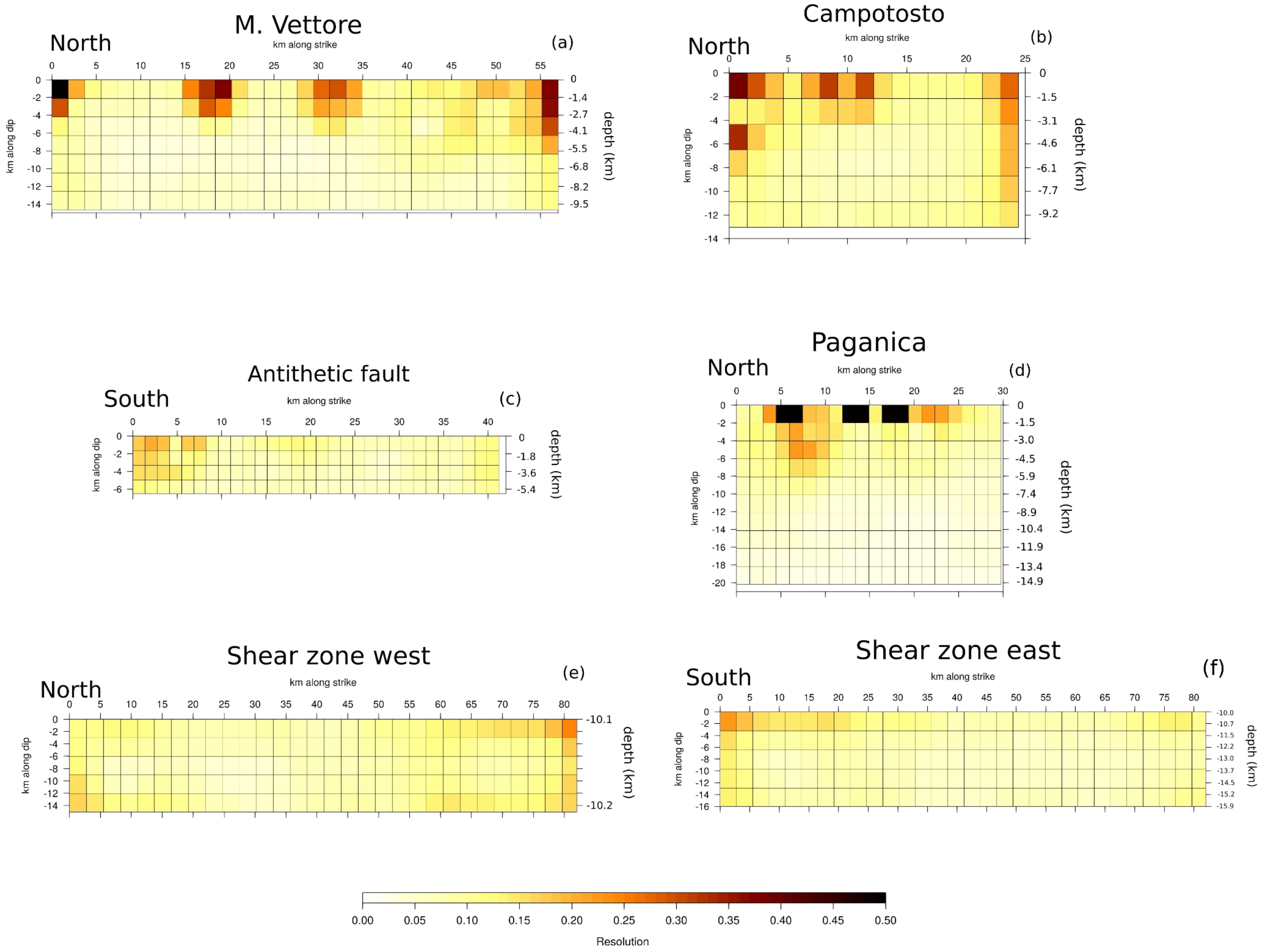

Fig S18. The resolution of the inversion of Section 4.3 main text for the M. Vettore fault (a), Campotosto fault (b), antithetic fault (c) , Paganica fault (d), western and eastern side of the shear zone (e) and (f).

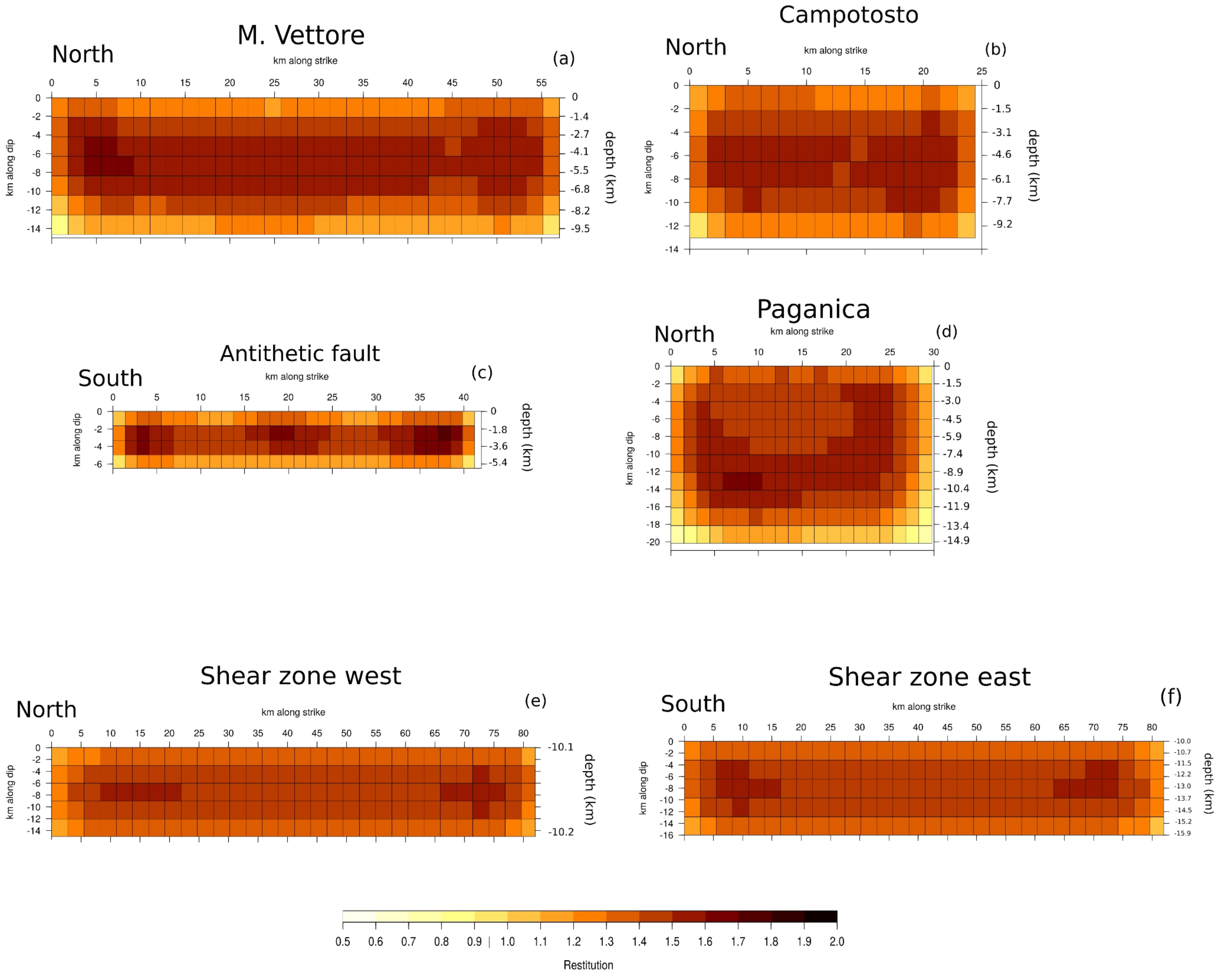

Fig S19. The restitution of the inversion of Section 4.3 main text for the M. Vettore fault (a), Campotosto fault (b), antithetic fault (c) , Paganica fault (d), western and eastern side of the shear zone (e) and (f).

S10. Coulomb Failure Function variation

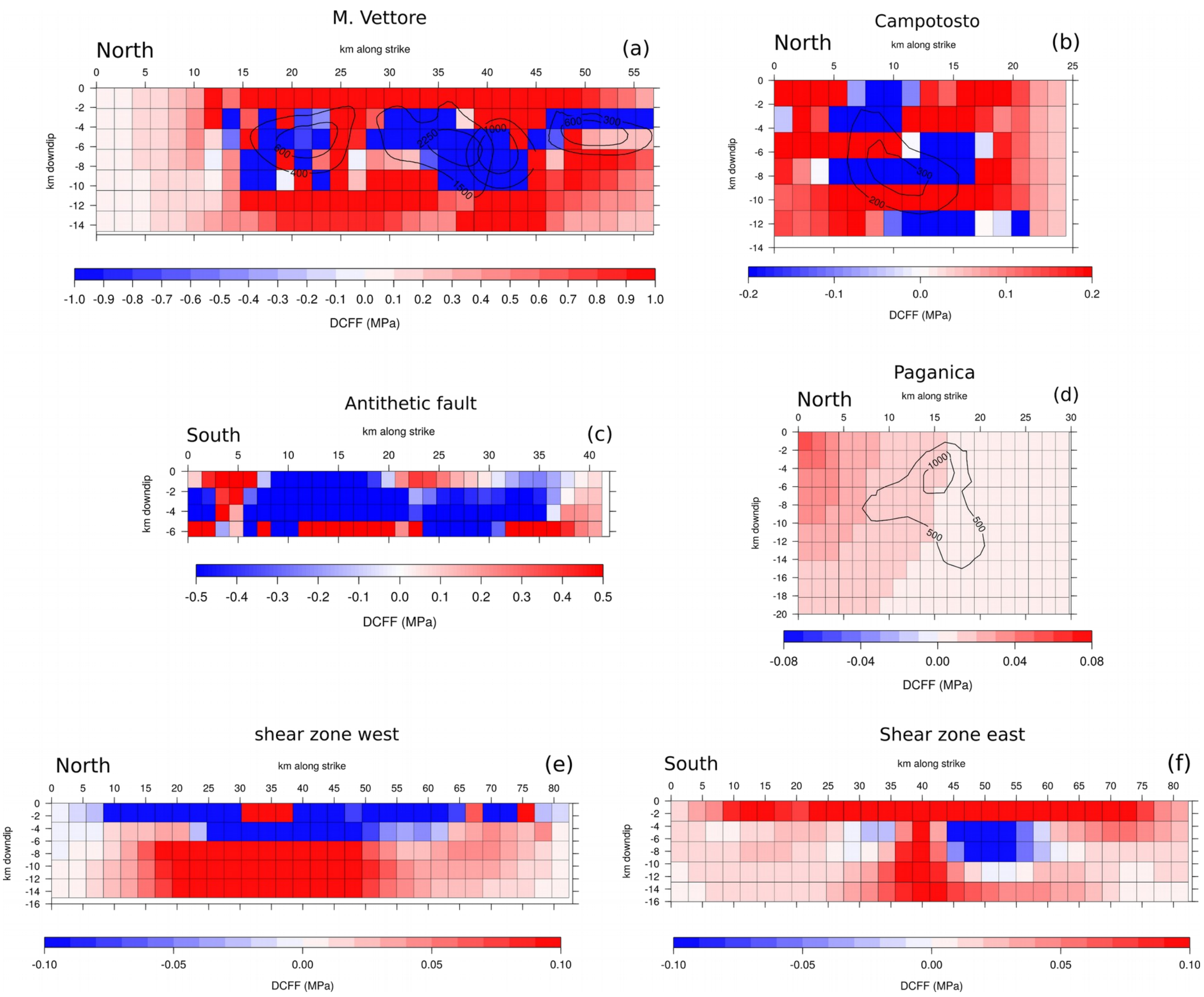

Fig S20. The Coulomb failure function variation (DCFF) on the M. Vettore fault (a), Campotosto fault (b), antithetic fault (c) , Paganica fault (d), western and eastern side of the shear zone (e) and (f), related to the main events of the 2016-2017 sequence (Section 1, main text) as modeled by Cheloni et al. (2017, 2019, 2019a). Co-seismic contours are as Figure 9 of maintext. Note the different scale below each fault plane (units of MPa).
